# Protective effect of the curcumin-baicalein combination against macrovascular changes in diabetic angiopathy

**DOI:** 10.3389/fendo.2022.953305

**Published:** 2022-08-18

**Authors:** Chenxiang Wang, Yibin Sun, Wenjing Liu, Yang Liu, Sualiha Afzal, Jahnavi Grover, Dennis Chang, Gerald Münch, Chun Guang Li, Shiling Lin, Jianyu Chen, Yiping Zhang, Zaixing Cheng, Yanxiang Lin, Yanfang Zheng, Mingqing Huang, Xian Zhou

**Affiliations:** ^1^ Fujian Key Laboratory of Chinese Materia Medica, College of Pharmacy, Fujian University of Traditional Chinese Medicine, Fuzhou, China; ^2^ NICM Health Research Institute, Western Sydney University, Westmead, NSW, Australia; ^3^ School of Medicine, Western Sydney University, Campbelltown, NSW, Australia; ^4^ Third Institute of Oceanography, Technical Innovation Center for Utilization of Marine Biological Resources, Ministry of Natural Resources, Xiamen, China

**Keywords:** curcumin, baicalein, endothelial dysfunction, diabetic angiopathy, synergy, network pharmacology, Nrf2-HO-1, MAPK/JNK

## Abstract

Endothelial dysfunction is an early pathological event in diabetic angiopathy which is the most common complication of diabetes. This study aims to investigate individual and combined actions of Curcumin (Cur) and Baicalein (Bai) in protecting vascular function. The cellular protective effects of Cur, Bai and Cur+Bai (1:1, *w/w*) were tested in H_2_O_2_ (2.5 mM) impaired EA. hy926 cells. Wistar rats were treated with vehicle control as the control group, Goto-Kakizaki rats (n=5 each group) were treated with vehicle control (model group), Cur (150 mg/kg), Bai (150 mg/kg), or Cur+Bai (75 mg/kg Cur + 75 mg/kg Bai, OG) for 4 weeks after a four-week high-fat diet to investigate the changes on blood vessel against diabetic angiopathy. Our results showed that Cur+Bai synergistically restored the endothelial cell survival and exhibited greater effects on lowering the fasting blood glucose and blood lipids in rats comparing to individual compounds. Cur+Bai repaired the blood vessel structure in the aortic arch and mid thoracic aorta. The network pharmacology analysis showed that Nrf2 and MAPK/JNK kinase were highly relevant to the multi-targeted action of Cur+Bai which has been confirmed in the *in vitro* and *in vivo* studies. In conclusion, Cur+Bai demonstrated an enhanced activity in attenuating endothelial dysfunction against oxidative damage and effectively protected vascular function in diabetic angiopathy rats.

## Introduction

As the major regulator of vascular homeostasis, the endothelium is an important locus to control vascular constriction and dilation (1). Endothelial dysfunction, characterised as a disrupted balance between vasodilation and vasoconstriction, is associated with many diseases including diabetes mellitus (DM), cardiovascular diseases and metabolic syndrome (2).

Endothelial injury occurs due to many risk factors including oxidative stress, inflammation, long-term hyperglycemia, insulin resistance, glucose and lipid metabolism disorders (3). In particular this process can result in the development of diabetic angiopathy, which is closely related to the mortality and morbidity arising from DM (4).

Hyperlipidemia is an independent risk factor of endothelial dysfunction. Chronic hyperglycemia and hyperlipidemia in DM both induce higher oxidative stress and inflammation in endothelium leading to excessive apoptosis and dysfunction of vascular endothelial cells. Cell damage increases the permeability of the endothelial layer, which promotes the invasion and accumulation of lipids in the blood vessel wall (5, 6). Additionally, immune cells such as monocytes are also recruited into the vascular endothelium due to inflammation, further contribute to the development of diabetic angiopathy.

Curcumin (Cur), the main natural polyphenol found in *Curcuma longa* L. species, has been demonstrated to markedly attenuate DM-induced endothelial dysfunction in animal studies (7, 8). The mechanism of Cur in protecting the endothelium have been investigated in the diabetic model. The benefit was mainly attributed to the inhibition of oxidative stress, as evidenced by decreased reactive oxygen species (ROS) (8), superoxide overproductions and increased heme oxygenase 1 (HO-1) activity (7). The efficacy of Cur in clinical trials remains inconclusive as it has been limited by poor bioavailability (low absorption and rapid excretion). Thus, research focus has been shifted to the modification of structure or the combined use of Cur with other biomolecules or phytochemicals to enhance its efficacy and bioavailability (9). For example, a Cur and vitamin C combination enhanced the effectiveness in protecting endothelial function, which was related to the strengthened antioxidant with hypoglycemic and hypolipidemic actions (10).

Baicalein (Bai) is a phytochemical isolated from the roots of *Scutellaria baicalensis* Georgi and *Scutellaria lateriflora* L. and possesses a multitude of pharmacological activities (11). It prevented the exaggerated constriction of blood vessels in the insulin-resistance rat model with macro-vascular impairment (12). It also inhibited high glucose-induced vascular inflammation in human umbilical vein endothelial cells (HUVECs) and mouse models (13). However, relatively high doses (100-150 mg/kg) of Bai were used in animal studies, restricting its further investigation in human clinical trials. The mechanism of action of Bai was associated with the upregulation of antioxidant nuclear factor erythroid 2–related factor 2 (Nrf2) mediated antioxidant pathway and downregulating Nuclear factor kappa B (NF-κB)-mediated inflammatory pathways (12, 13).

Chinese herbal medicines serve as an abundant source of drug discovery and development. The key mechanism of Chinese herbal medicines relies on the multi-component and multi-targeted action of the bioactive molecules. Thus, the optimal combination of selected bioactive molecules from Chinese herbal medicines may provide an attractive alternative or adjunct therapy in contrast to the single-entity, single-targeted pharmaceuticals (14). Network Pharmacology, a systems biology approach, can be applied to identify the bioactive molecules in the Chinse herbal medicines, and predict their targeted genes and signaling pathways (15). The development of mathematical models for synergistic interactions has enabled increasing research to determine synergy in natural products leading to novel combination therapies. Among them, combination index (CI) is one of the most popular models to determine the synergistic effect of two or more agents acting on a specific pharmacological target (i.e. receptor, gene, protein) in comparison to the action of each individual agent (16).

Based on the individual effects and associated mechanisms of Cur and Bai in protecting endothelium against diabetes, we speculated that Cur and Bai, when used as a combination, may interact synergistically leading to strengthened antioxidant activity and multi-targeted effects. This study aims to investigate the individual and combined effects of Cur and Bai against macrovascular changes in diabetic angiopathy by protecting the endothelium and their underpinning mechanisms via network pharmacology analysis, *in vitro* and *in vivo* investigations.

## Materials and methods

### Network construction and network analysis

To analyse the possible interaction of Cur and Bai against relevant disease targets, a network was constructed using the terms “endothelial dysfunction”, “diabetic angiopathy” and “atherosclerosis and endothelial dysfunction”. Briefly, the target disease condition (i.e. endothelial dysfunction)-related genes were obtained using the Online Mendelian Inheritance in Man (OMIM) Database (http://www.omim.org/), GeneCards Database (https://www.GeneCards.org/) and DisGeNET Database (http://www.disgenet.org/home/) by searching the key words “endothelial dysfunction”. Duplicate targets were removed after collecting all the genes targets in the three databases. The 2-dimensional structures of Cur and Bai were obtained from PubChem (https://pubchem.ncbi.nlm.nih.gov/) and their individual structure was subjected to the PharmMapper (http://www.lilab-ecust.cn/pharmmapper/) to populate their relevant gene targets. Common gene targets by Cur and Bai and disease condition were input to Search Tool for the Retrieval of Interacting Genes/Proteins (STRING, URL: https://string-db.org/4) to generate the protein-protein interaction (PPI) network. Disconnected nodes were hidden in the default setting of the network. Based on the constructed PPI network for each compound, we then investigated the associated Gene Ontology (GO) including biological process, cellular component and molecular function that represent gene product properties through the Database for Annotation, Visualization and Integrated Discovery (DAVID) online enrichment analysis database (https://david.ncifcrf.gov/home.jsp). We also explored the Kyoto Encyclopedia of Genes and Genomes (KEGG) pathways from DAVID. The top 5-15 targets in each function were selected and input to Bioinformatics (http://www.bioinformatics.com.cn/) to conduct the enrichment analysis of KEGG pathways and GO enrichment analysis, and the terms with a p-value less than 0.05 were filtered for the subsequent network construction. The overlapping gene targets were integrated and uploaded to Cytoscape (v.3.7.2) to construct the interactive network of “signaling pathway-gene target-drug”. The most relevant gene targets and KEGG pathways for both Cur and Bai were shown in the final network. The workflow for the network pharmacology analysis is shown in **Supplementary Material 1A**.

### H_2_O_2_ induced oxidative stress and drug treatments in endothelial cells

Human cardiovascular endothelial cell line (EA. hy926) was purchased from American Type Culture Collection (ATCC, USA, ATCC®CRL-2922™). Cells were cultured in DMEM/Ham’s F12 (Lonza, Australia) supplemented with 10% fetal bovine serum (FBS, Sigma, Australia) and 100 U/mL of penicillin-streptomycin (Gibco BRL, Australia). The cells from passage 20 to 30 were grown in a 5% CO2-humidified incubator at 37°C until confluency for the bioassays. The reference compounds of curcumin (Cur, Cat.no. BP0421) and baicalein (Bai, Cat.no. BP0232) were purchased from Chengdu Biopurify (China) with their identities confirmed by the high-performance liquid chromatography and purity over 98% (**Supplementary Material 2A, B**). They were both dissolved in dimethyl sulfoxide (DMSO, Sigma, Australia) at 15 mg/mL. Cur+Bai combination was prepared by mixing Cur and Bai both at 15 mg/mL in DMSO at the ratio of 1:1, *w/w*. Then Cur, Bai and Cur+Bai were dissolved in serum-free DMEM to 15 μg/mL and diluted by 1:2 fold with the final concentration of DMSO at 0.1%.

We followed our previous published method (9, 17) to investigate the protective effects of Cur, Bai, and Cur+Bai on endothelial cells against the oxidative damage from hydrogen peroxide (H_2_O_2_). EA. hy926 cells (1 × 10^6^ cells/mL) were seeded with DMEM/Ham’s F12 supplemented with 10% FBS and 100 U/mL of penicillin-streptomycin in the 96 well Corning Costar plate (Sigma, Australia) overnight. The media was replaced with serum-free DMEM/Ham’s F12, and the cells were incubated with serial diluted Cur, Bai, or Cur+Bai (0.43-15 μg/mL) for 1 h before the addition of H_2_O_2_ (Sigma-Aldrich, Australia) at 2.5 mM to induce the oxidative damage. Gallic acid acts as an antioxidant inhibiting oxidative species and enhance cell survival in the presence of H_2_O_2_ at low concentration (18–21). Thus, it has been used as comparison reference in our *in vitro* assays. EA. hy926 cells were co-incubated with 8.51 μg/mL (50 μM) gallic acid with a two-fold serial dilution 1 h prior to the stimulation of H_2_O_2_.

### Cell viability assessment by Alamar Blue and caspase-3 assays

EA. hy926 cells were pre-treated with serial diluted Cur, Bai and Cur+Bai for 1 h, and stimulated with H_2_O_2_ (2.5 mM) overnight. The cell supernatant was then replaced with Alamar Blue (10 μg/mL) in phosphate buffered saline (PBS) for 2 h. The fluorescent absorbance of Alamar Blue was measured at 540 nm excitation and 590 nm emission using a microplate reader (BMG LABTECH FLUOstar OPTIMA, Mount Eliza, Victoria, Australia). The cells were lysed on ice for 10 min, and then subjected to a Caspase-3 assay kit (ABCAM, Australia, ab39401) for the measurement of cellular protein levels of caspase-3 following the protocol in Zhou et al. (9, 17). The absorbance was read on a microplate reader (BMG LABTECH FLUOstar Optima, Mount Eliza, Victoria, Australia) at a wavelength of 410 nm.

### Intracellular oxidative status measurements by ROS expression

The intracellular level of oxidative stress with or without treatments was measured by ROS expression according to the protocol of cellular ROS assay kit (ABCAM, Australia, ab113851) cited in (9, 17). Briefly, EA. hy926 cells were stained with 2′,7′-dichlorofluorescin diacetate (DCFDA) (20 μM) at 100 μL per well for 45 min at 37°C in the dark. The plate’s initial absorbance was recorded as A0 with an excitation at 455 nm and an emission at 535 nm in fluorescence mode. After washing with ice-cold PBS, the cells were treated with Cur, Bai, Cur+Bai at increasing concentrations (0.86–15 μg/mL) or tertbutyl hydroperoxide (TBHP, 50 μM, positive control) for 1 h followed by the stimulation of H_2_O_2_ for 30 min. The absorbance was measured again and recorded as A1. The fold change of ROS was calculated as A1 normalised to its corresponding A0 (A1/A0).

### Intracellular oxidative status measurements by Nrf2 luciferase, superoxide dismutases and nicotinamide adenine dinucleotide enzymatic activities

The Nrf2-mediated pathway was investigated for the cyto-protection of Cur+Bai against oxidative stress. MCF7 AREc32 cells were cultured in DMEM (10% FBS, 1% penicillin), which were donated by Professor Gerald Münch in School of Medicine, Western Sydney University. Cells were seeded in 96-well plates at a density of 1.0 ×106 cell/mL till confluency and then treated with 0.1% DMSO in serum-free media (blank control), increasing concentrations of Cur, Bai, Cur+Bai, and tertiary butylhydroquinone (TBHQ, positive control). The Nrf2 total luciferin was detected by luciferase assay with optimization (9). The luminescence was measured within 30 min at an excitation wavelength of 488 nm and an emission wavelength of 525 nm. The activation of Nrf2 was calculated by fold compared to the blank control.

For the superoxide dismutases (SOD) assays, EA. hy926 cells cultured in the T25 flasks (Sigma, Australia) were treated with 0.1% DMSO, Cur, Bai, or Cur+Bai at the concentrations of 3.25 and 7.50 μg/mL for 4 h, and the cells were harvested by the lysis buffer (0.1 M tris HCl, pH 7.4 containing 0.5% Triton X-100, 5 mM β-ME, 0.1 mg/mL PMSF) on ice for 10 min. The protein was collected by centrifugation at 14,000×*g* for 5 min at 4°C. The collected protein was then subjected to the measurement of SOD using the commercial kit from Sigma-Aldrich (Australia) according to the manufacturer’s protocol (9).

For the nicotinamide adenine dinucleotide (NAD+) assay, EA. hy926 cells (1.0 × 106 cell/mL) were seeded on a 96-well plate (Sigma, Australia) overnight for 120 μL per well to allow confluency. The cells were then treated with media with 0.1% DMSO, Cur, Bai, or Cur+Bai at 7.5 μg/mL for 24 h. The total cellular NAD production was measured using the NAD+/NADH cell-based assay kit (Cayman, Australia) as cited in Zhou et al. (9).

### High-fat diet induced hyperlipidaemia rats and drug intervention

Following the bioassays in endothelial cells, the effect of Cur+Bai against macrovascular changes in diabetic angiopathy was investigated in the spontaneous model of type 2 diabetes, the Goto–Kakizaki (GK) rat (22, 23).

All animal experiments were conducted with the approval of the Animal Care and Use Committee of Fujian University of Traditional Chinese Medicine (approval number: 2021068), and followed the Animal Research: Reporting of *In Vivo* Experiments (ARRIVE) guidelines and the National Institutes of Health guide for the care and use of Laboratory animals (NIH Publications No. 8023, revised 1978). Male Wistar rats and Goto-Kakizaki rats (12-week-old, 300 ± 20 g) were purchased from Cavens Biogle (Suzhou) Model Animal Research Ltd. Co. (Suzhou, no. SCXK-2018-0002). Animals were provided with food and water ad libitum under specific pathogen free conditions at the temperature of 24°C ± 1°C, with 12-h light/dark cycle and unlimited drinking water supply.

After 7 days of acclimation, Wistar rats were given normal diet and GK rats were given high-fat diet containing 10% refined lard, 1.5% cholesterol, 0.3% pig bile salts and 88.2% normal diet for 4 weeks (22). The changes of fasting blood glucose (FBG) and body weights were monitored once a week throughout the trial. After 4 weeks, the Wistar rats were allocated to the control group (n=5): oral gavage (OG, passage of a gavage needle into the esophagus) administered with 0.5% sodium carboxymethyl cellulose solution (CMC-Na) as the vehicle control; The GK rats were randomly allocated to four groups (1) The model group (n=5) rats were OG administered with 0.5% CMC-Na in 10 mL; (2) Cur treatment group (n=5) were OG administered with Cur (150 mg/kg) in 10 mL 0.5% CMC-Na; 3) Bai treatment group (n=5) were OG administered with Bai (150 mg/kg dissolved in 10 mL of 0.5% CMC-Na; 4) Cur+Bai treatment group (n=5) were OG administered with Cur+Bai (75 mg/kg Cur + 75 mg/kg Bai both dissolved in 10 mL of 0.5% CMC-Na). All the treatments were given for 4 weeks (24). CMC-Na was used to enhance the drug stability of Cur and Cur+Bai in the prepared suspensions (25). The treatments in each group were given at 9 AM once daily. At the end of treatment (week 8), the animals were fasted for 12 h and anesthesitised with 25% Ulatan (1.1 g/kg, IP injection) before the blood was collected from the abdominal aorta. The aortic arch and mid thoracic aorta were collected immediately. The serum samples were collected by centrifugation at 500 g for 10 min, and the supernatants were collected for analysis.

### Body weight, fasted blood glucose test, blood lipid level and related oxidative and inflammatory biomarkers

The FBG and body weight were recorded once a week after the intervention (week 4-8). In preparation, the animals were fasted for 9 h before their tail blood was collected and tested using blood glucose test strips measured by the glucometer (Roche, USA). The FBG and body weight measurements at each time point were conducted three times for each animal, and the averaged value was recorded.

The blood lipid biomarkers including total cholesterol (TCHO), triglyceride (TG), non-esterified fatty acids (NEFA), low-density lipoprotein cholesterol (LDL-C) were measured using commercial kits (Nanjing Jiancheng Bioengineering Institute, China). The oxidative stress biomarkers including superoxide dismutase (SOD), myeloperoxidase (MPO), NAD+/NADPH levels were measured using commercial kits (Nanjing Jiancheng Bioengineering Institute, China). The inflammatory biomarker, tumor necrosis factor-α (TNF-α) was measured using ELISA kit (BOSTER Biological Technology co. ltd, China).

### Hematoxylin and eosin staining and TUNEL staining of blood vessel

Pathological hematoxylin and eosin (H&E) staining was conducted following steps in previous studies (26, 27). Briefly, aortic arch and mid thoracic aorta blood vessel tissues were collected, fixed with 4% paraformaldehyde, and dehydrated with ascending concentrations of ethanol. The tissues were cleaned with xylene I (100% ethanol and pure fresh xylene solution 1:1) and xylene II (pure fresh xylene) solutions for 40 min, respectively. The tissues were embedded in paraffin for 48 h and sectioned into 5 μm thickness. Each section was perfused in xylene I and II again for 10 min to remove the wax. Then the sectioned tissues were soaked in descending ethanol. Then they were stained with hematoxylin and eosin. These stained sections were sealed, mounted using Permount™ media, imaged and analysed using a light microscope. The dewaxed tissue was also subjected to the TUNEL staining with DAPI for blue staining of the nucleus and apoptotic cells stained with green fluorescence.

### Western blot analysis

EA. hy926 cells and entire aorta wall tissues from rats were washed twice with ice-cold PBS. Whole cell protein lysates were prepared by a lysis buffer supplemented with protease inhibitor mixture (Cell Signaling Technology, USA) and quantified by the Pierce BCA protein kit (Thermo Fisher Scientific, Australia). Equal amounts of protein samples (10 mg/mL) from each group were subjected to sodium dodecyl sulfate polyacrylamide gel electrophoresis and the proteins were transferred to PVDF membranes (Thermo Fisher Scientific, Australia). After being blocked with 5% skim milk at room temperature for 1 h, the membranes were incubated with the following primary antibodies overnight at 4˚C: caspase-1 p20 (1:500, SantaCruz, sc-398715), NLRP3 (1:1000, Abcam, 263899), Nrf2 (1:1000, Cell Signaling Technologies, 86806S), HO-1 (1:1000, Cell Signaling Technologies, 12721S), eNOS (1:1000, Cell Signaling Technologies, 32027S), p-JNK (1:1000, Cell Signaling Technologies, 9255S), JNK (1:1000, Cell Signaling Technologies, 9252S), p-p38 (1:1000, Cell Signaling Technologies, 4511S) and p38 (1:1000, Cell Signaling Technologies, 8690S). β-actin and GAPDH were used as the internal controls (1:3000, Beijing TransGen, China, catalogue number: HC201 and HC301) in this study. Then, the blots were washed in PBST three times (three min at a time) and subsequently incubated with anti-rabbit or anti-mouse horseradish peroxidase-conjugated secondary antibody (Cell signaling Technologies, 7074s or Proteintech, SA000-1, USA) for 1.5 h. Two separate gels were used for the examination of the phosphorylated and total proteins. The images were taken by the ChemiDoc XRS plus imaging system (Bio-Rad, Hercules, CA). The intensity of the targeted bands was quantified by ImageJ software. The quantitative data was presented as the ratio of intensity of targeted protein to that of β-actin.

### Synergy determination and statistical analysis

CompuSyn (Biosoft, US) was used to analyse the synergistic/antagonistic interaction between Cur and Bai based on the median-effect equation and isobologram analysis (16). The specific measurement for the combination index (CI) value represents the interaction level, where CI < 1, CI = 1, and CI > 1 suggest synergistic, additive, and antagonistic interaction, respectively. The calculation of CI values was based on Chou-Talalay method and determined by CompuSyn (28).

The values of concentrations and their corresponding responses of individual or combined Cur and Bai from the cell viability assay were input to the CompuSyn program. The CI-Fa (fraction affected level) curves were than generated with the CI and Fa values regarding the synergistic/antagonistic interactions. The CI-Fa curves show the dynamic changes of the interaction (based on CI values) with the different levels of Fa (cell viability).

All statistics comparisons were performed using GraphPad Version 9 (US). The significance was analysed by one-way analysis of variance (ANOVA) with Tukey’s multiple comparisons test and Dunnett’s test as post-hoc test, or unpaired t test. The data was expressed as mean ± standard deviation with at least three individual experiments (n ≥ 3) or mean ± standard error of the mean (SEM) with n=5 rats per group. *P*<0.05 was considered statistically significant.

## Results

### Identification of common gene and KEGG targets by network construction and analysis

For endothelial dysfunction, GeneCards, DisGeNET and OMIM searches generated 1500, 716 and 185 gene targets, respectively. The pharmacological actions of Cur and Bai have been connected with 49 and 47 gene targets, respectively. After filtering, 20 and 22 common gene targets were screened for Cur and Bai against endothelial dysfunction. As shown in **Supplementary Material 1B**, the compound–candidate target (C-T) network was constructed for Cur and Bai against endothelial dysfunction which consisted of 291 edges 63 nodes. The key common gene targets included MAPK14, MAPK8, MAPK 10, NQO1, CDK2, ESR1, ESR2, F2, HSP90AA1, ADAM17 (degrees ranged from 12-23), with MAPK 14 ranked as the top gene (the highest degree).

We have listed the top 15 signaling pathways that were involved in the targets of Cur and Bai against endothelial dysfunction (**Supplementary Material 1C, D**). The top 3 signaling pathways of Cur are pathways in cancer, Foxo signaling pathway, and endocrine resistance (ranked by degree). The top three signaling pathways of Bai are Estrogen signaling pathway, Chemical carcinogenesis-receptor activation and Fluid shear stress and atherosclerosis. The top 3 targeted signaling pathways shared by both Cur and Bai against endothelial dysfunction were identified through the constructed network. These were Endocrine resistance (degree=32), Pathways in cancer (degree=19), and Fluid shear stress and atherosclerosis (degree=16). Among them, only the Fluid shear stress and atherosclerosis pathway, is categorized in cardiovascular diseases; thus, particular attention was paid to this pathway as shown in **Supplementary Material 1E**. The top 10 KEGG pathways that are affected by both Cur and Bai are shown in **Table 1**.

**Table 1:** KEGG pathways affected by both Cur and Bai against endothelial dysfunction.

Pathway	Category	P value
Pathways in cancer	Cancer: overview	0.0000000170229
Endocrine resistance	Drug resistance: antineoplastic	0.00000128102
Fluid shear stress and atherosclerosis	Cardiovascular disease	0.00000720725
Epithelial cell signaling in Helicobacter pylori infection	Infectious disease: bacterial	0.0000111191
Prolactin signaling pathway	Endocrine system	0.0000111191
Progesterone-mediated oocyte maturation	Endocrine system	0.0000494524
Lipid and atherosclerosis	Cardiovascular disease	0.0000595532
Relaxin signaling pathway	Endocrine system	0.00012373
Estrogen signaling pathway	Endocrine system	0.000160666

This KEGG pathway map contains two major components (sub-pathways); the anti-atherogensis and pro-atherogensis pathway. In the anti-atherogensis pathway, Nrf2 has a central role as its translocation switches on the downstream functional responses against atherosclerosis including antioxidant, anti-inflammatory, vasodilatory, and anti-thrombotic actions. In the pro-atherogensis pathway, the phosphorylation of p38 and JNK induces the activation of transcription factor AP-1. It then leads to a serial cascades’ response of inflammation, matric degeneration, and angiogenesis contributing to pro-atherogensis.

Further network pharmacology analysis for Cur and Bai against diabetic angiopathy and atherosclerosis/endothelial dysfunction was also constructed as shown in **Supplementary Material 3, 4**, respectively.

### Synergistic effect of Cur+Bai on restoring cell viability of EA. hy926 cells

**Figure 1:**
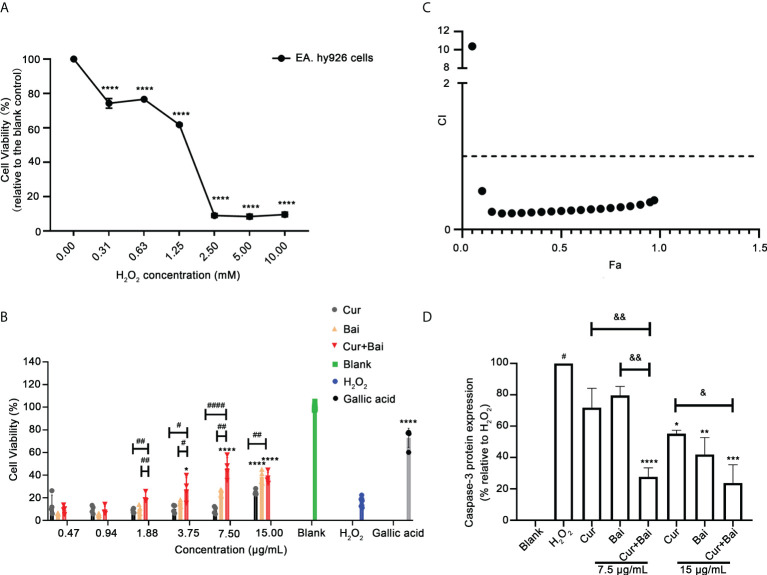
Synergistic activity of combined Cur and Bai in restoring H_2_O_2_ impaired cell viability in EA. hy926 cells assessed by Alamar Blue assays. **(A)** H_2_O_2_ dose-dependently reduced cell viability in EA. hy926 cells by Alamar Blue assay. **** *p*<0.0001 *vs.* H_2_O_2_ concentration = 0.00 mM in EA. hy926 cells. **(B)** Pretreatment of Cur, Bai, or Cur+Bai (1:1, *w/w*) restored the cell viability of EA. hy926 cells against the stimulation of H_2_O_2_ at 2.5 mM assessed by Alamar Blue assay. Gallic acid was used as a comparative reference. The effect of Cur+Bai in restoring the impaired cell viability was significantly greater than that of Cur or Bai at 1.62, 3.25, 7.50 and 15 μg/mL. *p<0.05, ****p<0.0001 *vs.* H_2_O_2_ only. ^#^ *p*<0.05, ^##^ *p*<0.01, ^####^ *p*<0.0001 *vs.* Cur or Bai individual treatment. **(C)** Synergistic interaction of Cur+Bai in restoring cell viability analysed by CI-Fa curve in EA. hy926 cells. Fa represents the cell viability, and CI<1 represents synergy whereas CI>1 represents antagonism. **(D)** Cur+Bai (7.5 and 15 μg/mL) exhibited an enhanced effect in inhibiting H_2_O_2_-induced caspase-3 protein expression in comparison to that of the individual compound. ^#^ *p*<0.05 *vs*. Blank (H_2_O_2_ = 0.00 mM), *p<0.05 *vs.* H_2_O_2_ (2.5 mM), ^&^ *p*<0.05, ^&&^ *p*<0.01 *vs.* Cur or Bai at the same concentration level. Results were expressed as mean ± standard deviation for at least three individual experiments. The *p* values were obtained from one-way ANOVA analysis.. **p<0.01, ***p<0.001, ****p<0.0001.

Following the results obtained from network pharmacology analysis, the individual and combined actions of Cur and Bai on EA. hy926 cells were explored to see if they exhibited the expected protective effect on endothelium against oxidative stress which is one of the risk factors in diabetic angiopathy. As shown in **Figure 1A**, H_2_O_2_ dose-dependently (0.31–10 mM) reduced the cell viability compared to the untreated cells (cells with media only, *p*<0.0001) assessed by the Alamar Blue assay. At 2.5 mM, H_2_O_2_ reduced the cell viability dramatically to 8.99 ± 1.11% only. Thus, this concentration of H_2_O_2_ was selected to establish the oxidative stress induced cell impairment model in EA. hy926 cells. As shown in **Figure 1B**, gallic acid restored the cell viability to 72.92 ± 8.54% at 2.12 μg/mL (12.46 μM) against the oxidative damage of H_2_O_2_.

In **Figure 1B**, the pretreatment with Bai significantly restored cell viability to 38.42 ± 5.80% at 15 μg/mL (*p*<0.0001), whereas Cur (0.47-15 μg/mL) showed a dose-dependent increasing trend of the cell viability but did not reach significance. Combinations of Cur+Bai showed a prominent effect in restoring cell viability in a dose-dependent manner. Particularly, Cur+Bai (3.75 – 15 μg/mL) showed significantly higher cell viabilities compared to that of the blank control (*p*<0.05), and the improvement was consistently higher than that of Cur or Bai alone (*p*<0.05). CI model in **Figure 1C** revealed a strong synergy (CI values< 0.53) in most concentration levels when Fa ranged from 0.1 to 0.97 (representing 10%-97% cell viability levels).

Following this, the activity of total intracellular caspase-3 enzymes was assessed to determine whether Cur+Bai reduced the apoptotic cells against the impairment from H_2_O_2_. As shown in **Figure 1D**, EA. hy926 cells incubated with H_2_O_2_ (2.5 mM) for 24 h caused a significant increase in caspase-3 activity measured at 24 h (*p*<0.05) compared to that of the blank control, indicating an elevated cell apoptotic level with the stimulation of H_2_O_2_. At concentration level of 7.5 μg/mL, the effect of Cur and Bai were insignificant, whereas Cur+Bai markedly reduced the caspase-3 level to a comparable level of blank control (*p*<0.0001). The reduction of caspase-3 by Cur+Bai was markedly greater than that of Cur or Bai (both *p*<0.01). At 15 μg/mL, all the treatments demonstrated a down-regulatory trend of caspase-3, with the lowest expression of caspase-3 protein by Cur+Bai (*p*<0.001) which was greater than that of Cur alone (*p*<0.05).

### Cur+Bai increased Nrf2-regulated antioxidant response and downregulated MAPK-JNK pathway in EA. hy926 cells

**Figure 2:**
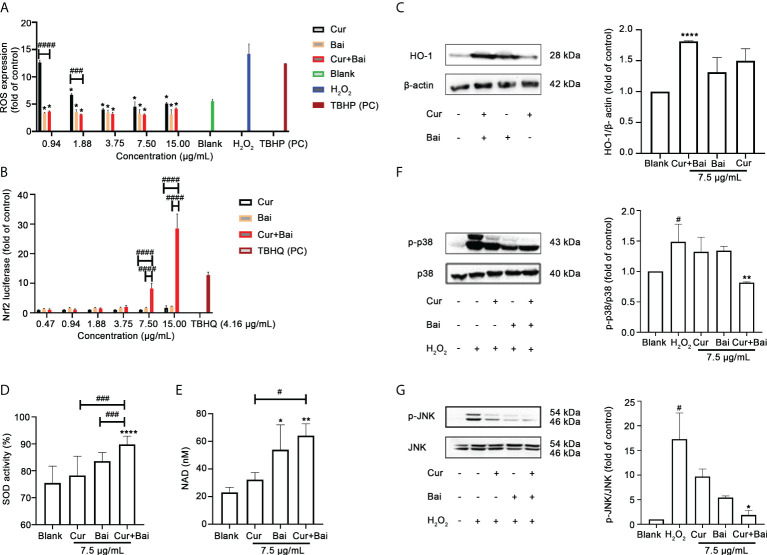
Cur+Bai attenuated H_2_O_2_-induced endothelial damage in EA. hy926 cells which may be associated with regulating Nrf2-HO-1 and MAPK pathways. **(A)** ROS expression in EA. hy926 cells pretreated with Cur, Bai or Cur+Bai with serial dilutions from 15 μg/mL and stimulated with H_2_O_2_ for 4 h. TBHP (50 μM) was used as the positive control. The ROS expression was presented as the fold change to the blank control (cells with media only). *p<0.05 *vs.* H_2_O_2_ only. ^###^ *p*<0.001, ^####^ *p*<0.0001 *vs.* Cur or Bai at the same concentration level. n=3 individual experiments. **(B)** Nrf2 luciferase under the treatment of Cur, Bai or Cur+Bai (0.47-15 μg/mL). The Nrf2 luciferase was presented as fold change to that of the blank control (cells with media only). TBHQ was used as the positive control. ^####^ *p*<0.0001 *vs.* Cur or Bai at the same concentration level. n=3 individual experiments. **(C)** The up-regulatory activity of Cur+Bai on HO-1/beta-actin which was greater than that of Cur or Bai alone (7.5 μg/mL) as assessed by the Western blot analysis (n=3 individual experiments). ****p<0.0001 *vs.* blank. Cur+Bai increased cellular SOD **(D)** and NAD (nM) **(E)** productions. *p<0.05, **p<0.01 *vs.* blank; ^#^ *p*<0.05, ^###^ *p*<0.001 *vs.* Cur or Bai at the same concentration level (7.5 μg/mL). The error bars represent the standard deviation of measurements for over three samples in three separate assay runs (n = 3). **(F, G)** Cur+Bai (7.5 μg/mL) downregulated the phosphorylation of MAPKp38 and JNK as assessed by the Western blot analysis (n=3 individual experiments). ^#^ *p*<0.05 *vs.* blank control. *p<0.05, **p<0.01 *vs.* H_2_O_2_. Results were expressed as mean ± standard deviation for at least three individual experiments. The *p* values were obtained from one-way ANOVA analysis.

**Figure 2A** shows that ROS increased by 14.21 ± 1.82 times in H_2_O_2_ (2.5 mM) stimulated EA. hy926 cells whereas non-treated cells only expressed 5.53 ± 0.32 fold of ROS. TBHP was used as a positive control which boosted the ROS level by 12.42 ± 0.32 fold. Treatment of Cur, Bai, and Cur+Bai significantly reduced the ROS fold change against H_2_O_2_ (2.5 mM) at 1.88–15 μg/mL (all *p*<0.05). Furthermore, Cur+Bai showed a significantly greater inhibition of ROS than that of Cur at 0.94 and 1.88 μg/mL (*p*<0.001) when compared at the same concentration level.

The activation of the Nrf2 activity by Cur+Bai was investigated to see if it was associated with the synergistic protective effect of Cur+Bai on endothelial cells against oxidative damage as indicated by the network pharmacology analysis. As shown in **Figure 2B**, Cur+Bai dose-dependently increased the Nrf2 luciferase with the maximum fold change of 28.48 ± 4.91 at 15 μg/mL, whereas the up-regulatory effect by Cur or Bai alone were insignificant. The positive control, TBHQ, increased Nrf2 expression by 12.76 ± 1.01 fold at 4.16 μg/mL (25 μM). We then analysed the induction of HO-1 protein by Cur, Bai, or Cur+Bai, as the downstream protein target regulated by Nrf2 (**Figure 2C**). Individual Cur and Bai at 15 μg/mL showed an increasing trend of HO-1 expression compared to that of untreated cells, however, the increase did not reach a significance level. Cur+Bai (15 μg/mL) increased the HO-1 protein expression by 1.81 ± 0.01 times with the greatest increase (*p*<0.0001) among three treatments.

The expressions of SOD and NAD+ were examined as Nrf2-HO-1 regulated antioxidant (co)enzymes (**Figure 2D, E**). Cur or Bai alone at 7.5 μg/mL showed an increasing trend of SOD activity, although it did not reach a statistical significance compared to that of untreated cells (blank). In contrast, Cur+Bai (7.5 μg/mL) significantly increased the SOD activity (89.84 ± 3.04% *vs*. blank 75.52 ± 6.23%, *p*<0.0001). The increase of SOD activity by Cur+Bai was significantly higher than that of Cur or Bai (both *p*<0.001). Similarly, Cur+Bai (7.5 μg/mL) significantly increased the total intracellular NAD production compared to that of the blank control (untreated cells) (64.20 ± 8.51 nM *vs*. blank 23.08 ± 3.63 nM, *p*<0.01). The increase of NAD by Cur+Bai (7.5 μg/mL) was significantly higher than that of Cur (*p*<0.05) but comparable to that of Bai alone.

Western blot analysis was conducted to analyse the regulated phosphorylation of MAPKp38 and JNK by Cur+Bai, Cur or Bai. Our results in **Figures 2F, G** showed that the stimulation of H_2_O_2_ (2.5 mM) significantly upregulated the phosphorylation of MAPKp38 and JNK by 1.49 ± 0.29 and 17.31 ± 9.19 times, respectively compared to the blank control (both *p*<0.05). The treatment of Cur or Bai (7.5 μg/mL) did not show a significant inhibition of p-p38/p38 whereas Cur+Bai (7.5 μg/mL) significantly reduced the expression (*p*<0.01 *vs*. H_2_O_2_) to a comparable level of the blank control. Cur+Bai (7.5 μg/mL) also demonstrated a significant downregulation of p-JNK/JNK to 1.91 ± 0.61 fold change compared to that of H_2_O_2_ only (*p*<0.05), whereas the effects of Cur or Bai (7.5 μg/mL) were less remarkable.

### Cur+Bai protected aortic blood vessels and decreased the blood lipid levels in diabetic rats

The individual and combined effects of Cur and Bai on endothelial and vascular function in GK rat model were investigated.

**Figure 3:**
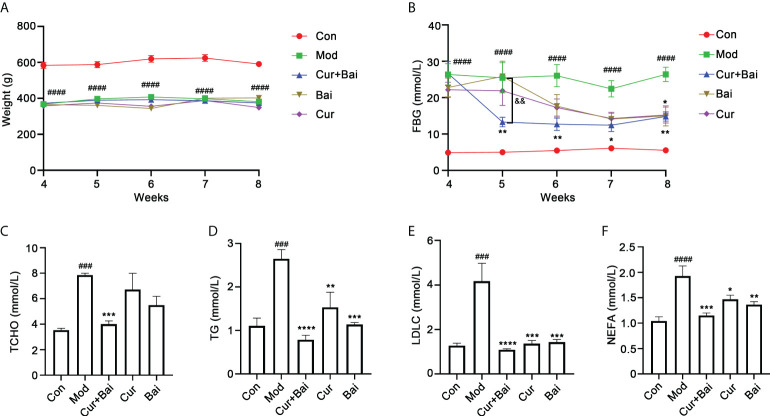
Cur+Bai effectively lowered the FBG and blood lipid levels in diabetic rats. **(A)** The body weights of diabetic model group were constantly lower than that of the control group (n=5 per group). The treatments of Cur+Bai, Cur or Bai did not change body weight from week 4 to week 8 compared to that of the diabetic model group. ^####^ *p*<0.0001 *vs.* control group. **(B)**. Cur+Bai, Cur and Bai time-dependently reversed the HFD-induced high FBG level (mmol/L) in diabetic group (n=5 per group) from week 4 to week 8. The FBG lowering effect in the Cur+Bai was greater than that of Cur or Bai group from week 5 to week 7. ^####^ *p*<0.0001 *vs.* Con at the same time point. *p<0.05, **p<0.01 *vs.* Mod at the same time point. ^&&^ *p*<0.01, Cur+Bai *vs.* Bai compared at the same time point. The changes of blood lipid levels by Cur+Bai, Cur and Bai in the diabetic rats, including TCHO **(C)**, TG **(D)**, LDLC **(E)** and NEFA **(F)** with n=5 per group. ^###^ *p*<0.001, ^####^ *p*<0.0001 *vs.* Con at the same time point. *p<0.05, **p<0.01, ***p<0.001, ****p<0.0001 *vs.* Mod at the same time point. Results were expressed as mean ± SEM for five rats per group. The *p* values were obtained from one-way ANOVA analysis.

The changes in blood glucose level and body weight were monitored every week from week 4 to week 8. As shown in **Figure 3A**, the body weight in the control group remained at a similar level throughout the trial, with an average weight of 583.4 ± 37.6 g in week 4 to 591.0 ± 31.0 g in week 8. In comparison, the body weight in the GK rat model group was averaged at 367.8 ± 12.8 g in week 4 and 381.6 ± 11.0 g in week 8. The treatment of Cur, Bai or Cur+Bai did not show significant change of the body weight compared to the model group throughout the trial.

As shown in **Figure 3B**, the FBG level in the model group was constantly higher than that of the control group from week 4 (26.37 ± 6.65 *vs.* 4.92 ± 0.54 mmol/L, *p*<0.0001) to week 8 (26.42 ± 4.43 *vs.* 5.56 ± 0.34 mmol/L, *p*<0.0001). The treatment of Cur+Bai markedly reduced the FBG level starting from week 5 (13.30 ± 2.97 mmol/L), which was significantly lower than that of the model group (25.49 ± 9.28 mmol/L, *p*<0.05). Moreover, the FBG reducing effect of Cur+Bai was greater than that of Bai (25.85 ± 9.41 mmol/L, *p*<0.01) or Cur alone (21.91 ± 9.08 mmol/L, *p*<0.01) at week 5 which the individual effects were insignificant. The individual effects of Cur and Bai were not obvious until week 8 which reached a statistical significance with *p*<0.05. In contrast, Cur+Bai constantly lowered FBG level from week 6 to week 8 (*p*<0.05) with the FBG level ranging from 12.46 ± 3.91 to 14.86 ± 2.85 mmol/L.

The blood lipid levels were measured using the serum collected from the abdominal aorta. As shown in **Figures 3C–F**, the model group showed significantly higher levels of TCHO (*p*<0.001), TG (*p*<0.001), LDLC (*p*<0.001) and NEFA (*p*<0.0001) in comparison to that of the control group. Cur alone did not show a significant effect in lowering TCHO, but markedly reduced TG (*p*<0.01), LDL-C (*p*<0.001) and NEFA (*p*<0.001). Similarly, Bai was not effective in lowering TCHO, but significantly reduced TG (*p*<0.001), LDLC (*p*<0.001), NEFA (*p*<0.01). Remarkably, the treatments of Cur+Bai constantly showed significant effects in reducing their levels (all p values<0.001). Moreover, the greatest reductions were generally seen in Cur+Bai for all the tested blood lipids compared to that of Cur or Bai alone, although no significance difference was detected.

**Figure 4:**
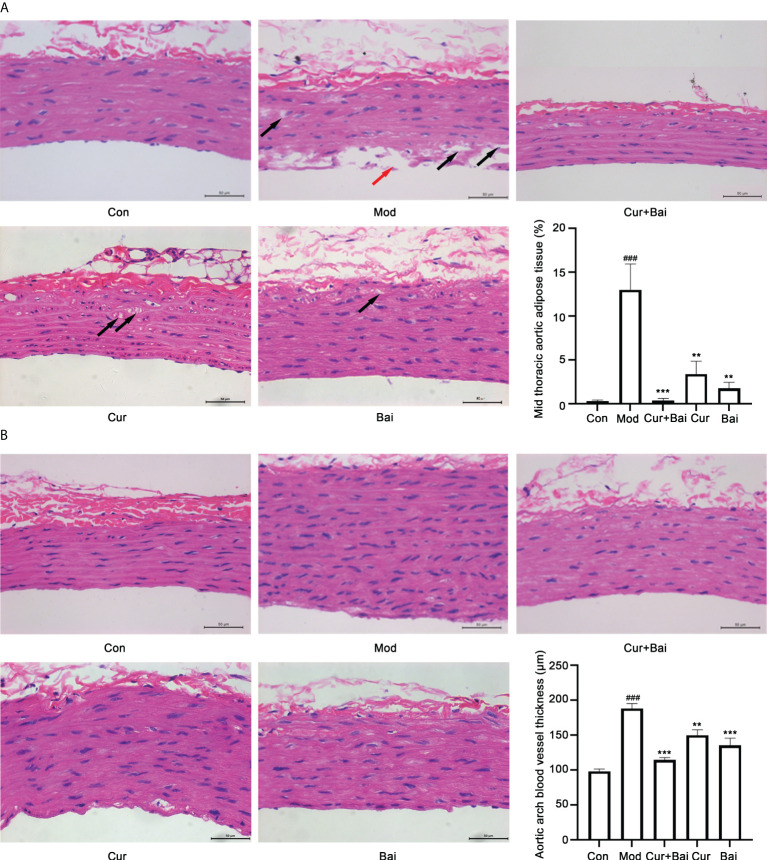
The treatments of Cur+Bai, Cur and Bai repaired the blood vessel morphology in both mid thoracic and aortic arch tissue in the diabetic rats. **(A)** Representative pathological staining of blood vessel tissue in the mid thoracic aorta in the control, model, Cur+Bai, Cur and Bai treated groups (n=5 per group). Scale bar = 50 um. Red arrow points to the injured inner surface of the blood vessel. Black arrows point to the adipose tissue in the middle layer of the blood vessel. The statistical analysis on the mid thoracic aorta adipose tissue (percentage) in five groups. Dynamic Med 6.0 microscopic image analysis system was used for the quantitative analysis of adipose tissue in the aorta. The adipose tissue in the aorta (%) was calculated as below: Aorta adipose tissue (%) = the sum of adipose tissue areas in the H&E staining area/total area of the blood vessel in the same H&E staining area* 100%. ^###^ *p*<0.001 *vs.* Con group. **p<0.01, ***p<0.001 *vs.* Mod group. **(B)** Representative pathological staining of blood vessel tissue in the aortic arch in the control, model, Cur+Bai, Cur and Bai treated groups (n=5 per group). Scale bar = 50 um. The statistical analysis on the thickness of the aortic arch blood vessel wall (μm) in five groups. ^###^ *p*<0.001 *vs.* Con group. **p<0.01, ***p<0.001 *vs.* Mod group. Results were expressed as mean ± SEM for five rats per group. The *p* values were obtained from one-way ANOVA analysis.

The pathological changes of blood vessels in the mid thoracic and aortic arch tissues by the treatment of Cur+Bai were monitored. As shown in **Figure 4A**, a smooth inner surface of the blood vessel was observed in the control group, whereas the model group showed a damaged inner surface of the blood vessel (indicated by a red arrow) and the presence of lipid droplets (adipose tissue, indicated by black arrows) in the middle layer of the blood vessel. The treatment of Cur+Bai showed a normal morphology of the blood vessel with a smooth surface of the inner layer and very few adipose tissues in the middle layer. Both the individual treatments of Cur and Bai showed smooth endothelium layer, however, adipose tissues were observed especially in the Cur-treated group. The statistical analysis showed a significantly higher percentage of aortic adipose tissue in the model group (13.01 ± 6.55%) compared to that of the control group (0.28 ± 0.31%, *p*<0.001). The treatments of Cur+Bai (0.36 ± 0.51%, *p*<0.001), Cur (3.40 ± 3.23%, *p*<0.01) and Bai (1.72 ± 1.56%, *p*<0.01) all significantly lowered the adipose tissue level compared to that of the model group. The lowest adipose tissue (%) was shown in the Cur+Bai treated group. In addition, as shown in **Figure 4B**, it was quite evident that the blood vessel wall was much thicker in the model group compared to that of the control group (*p*<0.001). The treatment with Cur+Bai (*p*<0.001), Cur (*p*<0.01) or Bai (*p*<0.001) all significantly reduced the blood wall thickness compared to that of the model group.

**Figure 5:**
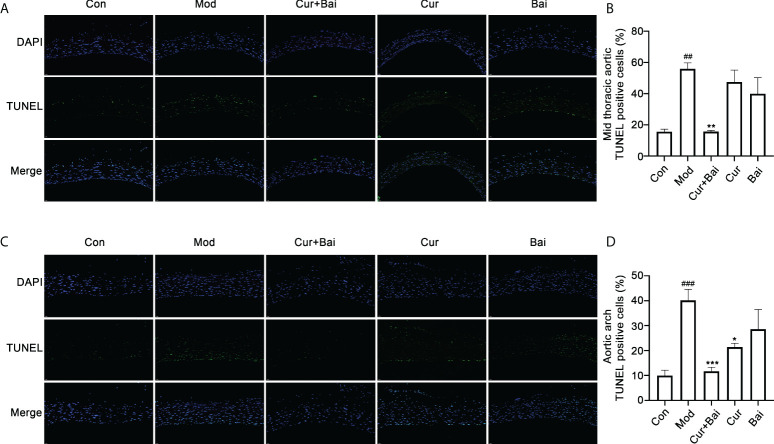
TUNEL staining for the mid thoracic and aortic arch blood vessel tissue in the control, model Cur+Bai, Cur and Bai treated groups. Represent TUNEL staining images of blood vessel in thorax **(A)** and heart **(B)**. Statistical analysis of the TUNEL positive cells in the mid thoracic **(C)** and aortic arch **(D)**. ^##^ *p*<0.01, ^###^ *p*<0.001 *vs.* control group. *p<0.05, **p<0.01, ***p<0.001 *vs.* model group. Results were expressed as mean ± SEM for five rats per group. The *p* values were obtained from one-way ANOVA analysis.

The effect of Cur+Bai in restoring cell survival in the blood vessels was examined by the TUNEL staining. **Figures 5A, C** revealed that there was obvious cell apoptosis in blood vessel (as highlighted by the green fluorescence) in both mid thoracic aorta and aortic arch. The statistical analysis in **Figures 5B, D** showed the significant higher TUNEL positive cells % in the model group in the mid thoracic aorta and aortic arch (*p*<0.01 and *p*<0.001). There were very few apoptotic cells in the Cur+Bai treated groups as evidenced by the weak green fluorescence and significantly lower TUNEL positive cells % (mid thoracic aorta: *p*<0.01 and aortic arch: *p*<0.001). Cur significantly reduced the TUNEL positive cells in the aortic arch (*p*<0.05), whereas Bai was insignificant in the aortic arch or mid thoracic aorta.

### Cur+Bai increased the eNOS protein expression, protected aorta blood vessels in relation to Nrf2-regulated antioxidant enzymes and MAPK pathway

**Figure 6:**
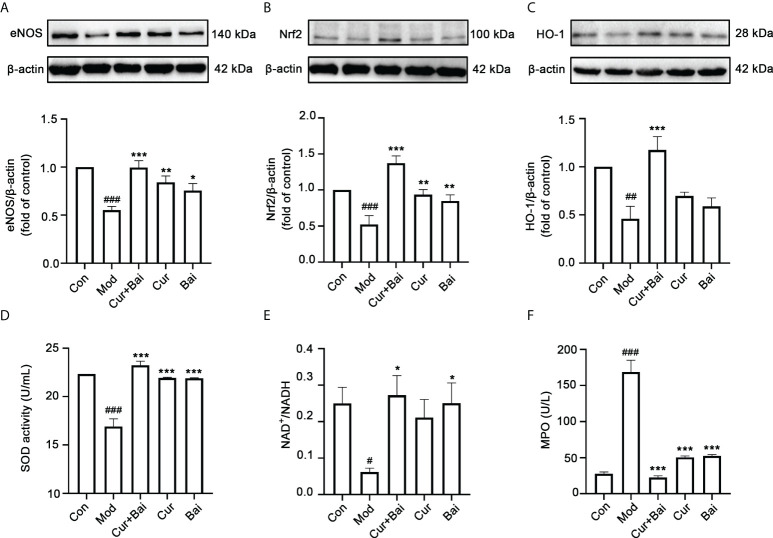
The treatment of Cur+Bai upregulated eNOS protein expression, Nrf2-HO-1 mediated antioxidant activity and downregulated MAPKp38-JNK pathway in the entire aorta wall in the diabetic rats. **(A)** Cur+Bai, Cur and Bai upregulated eNOS protein expression in diabetic rats as analysed by the Western blot analysis. n=3, ^###^ *p*<0.001 *vs.* control group, *p<0.05, *p*<0.01, *p*<0.001 *vs.* model group. **(B)** Cur+Bai, Cur and Bai upregulated Nrf2 protein expression in diabetic rats as analysed by the Western blot analysis. n=3, ^###^ *p*<0.001 *vs.* control group, **p<0.01, ***p<0.01 *vs.* model group. **(C)** Cur+Bai upregulated HO-1 protein expression in diabetic rats as analysed by the Western blot analysis. n=3, ^##^ *p*<0.01 *vs.* control group, ***p<0.001 *vs.* model group. **(D)** Cur+Bai, Cur and Bai upregulated the SOD enzymatic activity (U/mL). n=5 per group, ^###^ *p*<0.001 *vs.* control group, ***p<0.001 *vs.* model group. **(E)** Cur+Bai and Bai promoted the NAD+/NADPH ratio. n=5 per group, ^#^ *p*<0.05 *vs.* control group, *p<0.05 *vs.* model group. **(F)** Cur+Bai, Cur and Bai downregulated the MPO expression (U/mL) in the diabetic rats. n=5 per group, ^#^ *p*<0.05 *vs.* control group, *p<0.05 *vs.* model group. Results were expressed as mean ± SEM for five rats per group. The *p* values were obtained from one-way ANOVA analysis.

In diabetes, the expression of endothelial nitric oxide synthase (eNOS) is altered which leads to endothelial dysfunction and the progression of diabetic angiopathy (29). The changes in eNOS protein expression were examined by Western Blot analysis as shown in **Figure 6A**. The model group showed a significantly lower eNOS protein expression (*p*<0.001) compared to the control group, suggesting an impaired endothelial function in the diabetic animals. On the other hand, treated groups including Cur+Bai, Cur and Bai all significantly reversed the eNOS protein expressions (*p*<0.05), with Cur+Bai showed the highest expression of eNOS (*p*<0.001).

The Nrf2-mediated antioxidant defensive mechanism by Cur+Bai was investigated *in vivo*. As shown in **Figures 6B, C**, the protein levels of Nrf2 (*p*<0.001) and HO-1 (*p*<0.01) were significantly lower in the diabetic model group compared to that of the control group. The treatment of Cur+Bai significantly restored the Nrf2 (*p*<0.001) and HO-1 (*p*<0.001) protein expressions in comparison to that of the model group. Individual Cur and Bai also significantly increased the Nrf2 protein expression (*p*<0.01), however, they were insignificant in restoring the protein expression of HO-1. Following that, Cur+Bai significantly restored the expressions of SOD (*p*<0.001) and NAD+/NADH (*p*<0.05) compared to that of the model group (**Figures 6D, E**) which was consistent to that of the *in vitro* findings. Individual Cur and Bai significantly increased SOD expression (*p*<0.001) to the same extend as the Cur+Bai combination. Bai also increased the level of NAD+/NADH (*p*<0.05) whereas Cur did not show any significant effect. Cur+Bai significantly reduced the expression of MPO (*p*<0.001) compared to that of the model group (**Figure 6F**). The combined effect was comparable to that of Cur and Bai alone.

**Figure 7:**
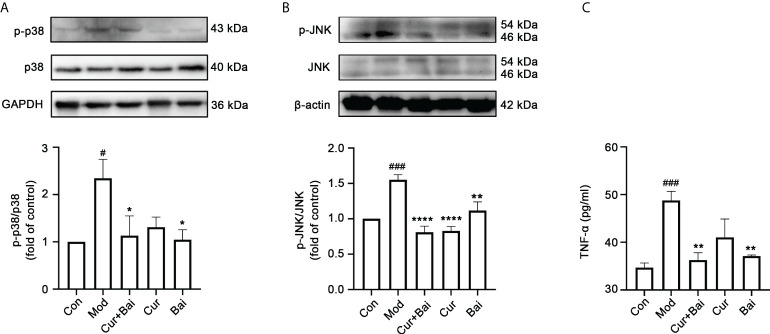
The treatment of Cur+Bai downregulated phosphorylated MAPKp38 and JNK, and reduced the production of TNF-α in the entire aorta wall in the diabetic rats. **(A)** Cur+Bai and Bai downregulated phosphorylated MAPKp38 expression in diabetic rats as analysed by the Western blot analysis. n=3, ^#^ *p*<0.05 *vs.* control group, *p<0.05 *vs.* model group. **(B)** Cur+Bai, Cur and Bai downregulated phosphorylated JNK expression in diabetic rats as analysed by the Western blot analysis. n=3, ^###^ *p*<0.001 *vs.* control group, **p<0.01, ****p<0.0001 *vs.* model group. **(C)** The treatment of Cur+Bai downregulated the TNF-α production expression in the diabetic rats. n=5, ^###^ *p*<0.001 *vs.* control group, **p<0.01 *vs.* model group. Results were expressed as mean ± SEM for five rats per group. The *p* values were obtained from one-way ANOVA analysis.

Since both the network pharmacology analysis and *in vitro* testing showed that p-p38 and p-JNK could be the key targets of Cur+Bai, these two protein targets were tested *in vivo*. As shown in **Figures 7A, B**, the phosphorylated p38 (*p*<0.05) and JNK (*p*<0.001) were significantly increased in the model group. In contrast, the treatment of Cur+Bai significantly lowered the expressions of p-p38 (*p*<0.05) and p-JNK (*p*<0.0001), suggesting that MAPKp38 and JNK still play a role in the action of Cur+Bai in protecting blood vessel and endothelium in diabetic animals. The downregulation of p-p38 by Cur+Bai was comparable to that of Bai alone (*p*<0.05 *vs.* model group), whereas Cur showed insignificant effect. Interestingly, Cur significantly reduced p-JNK (*p*<0.0001) which was similar to that of Cur+Bai, and Bai also showed a significant inhibition (*p*<0.01).

The reduced level of MPO led to the investigation of inflammatory markers in the blood vessel by Cur+Bai. As shown in **Figure 7C**, the level of TNF-α was significantly elevated in the model group (*p*<0.001) and the treatment of Cur+Bai significantly lowered the level (*p*<0.01) which was at a comparable level to that of the control group. Bai also significantly reduced TNF-α production (*p*<0.01), however, the effect from Cur was insignificant.

## Discussions

Endothelial dysfunction is an early marker in the pathogenesis of vascular diseases (30). It also has a strong link with diabetic angiopathy which is the major complication of DM. Many risk factors of DM are involved in the development of endothelial dysfunction such as hyperglycaemia and hyperlipidaemia that cause oxidative stress and inflammation in endothelium. Cur and Bai are two nutraceuticals that have been individually demonstrated to protect the endothelium in DM through antioxidant and anti-inflammatory related pathways. The present study investigated the combined activity of Cur and Bai in rescuing endothelial survival against the impairment of H_2_O_2_ in EA. hy926 cells, and protecting blood vessels in aortic arch and mid thoracic aorta of diabetic rats. The detailed mechanisms of the Cur and Bai combination were elucidated through the network pharmacology analysis and partially validated by the *in vitro* and *in vivo* experimental investigations. To our knowledge, this is the first study that investigates the enhanced pharmacological activity of a Cur and Bai combination by in silico analysis, cellular assays and animal models.

The potent antioxidant activity of Cur has led to a few studies that investigated its individual effect in protecting the endothelium against a variety of impairments (31). A study from Sun etal. (32) suggested that the pretreatment of Cur (25 μM) partly inhibited the H_2_O_2_ induced oxidative stress, and attenuated H_2_O_2_-induced apoptosis index (9.67 ± 1.26% *vs*. 15.00 ± 1.77%, *p*<0.05) in HUVECs (32). Bai was shown to mitigate endothelial injury against radiation-induced enteritis and inhibit lipopolysaccharides-induced proinflammatory response and apoptosis in HUVECs (33, 34). Based on these previous studies, we tested the pharmacological activities of Cur, Bai and their combination using a generic and common endothelial injury model induced by H_2_O_2_. EA. hy926 cells are the human umbilical vein cell line which was established by fusing primary human umbilical vein cells with a thioguanine-resistant clone of A549 by exposure to polyethylene glycol (PEG). This cell line has been widely used in various studies on endothelial cells and blood vessel related research. Previous studies have demonstrated that EA. hy926 preserves similar characteristics with primary human endothelial cells, such as Human Aortic Endothelial Cells (HAEC) (35, 36). Exposure to hydrogen peroxide ( H_2_O_2_) is widely used procedure to cause oxidative damage/stress in cellular models. The Fenton’s reaction between H_2_O_2_ and Fe2+ ions generates the highly reactive OH radical and is thought to be the main mechanism for oxidative damage (37). Many studies have applied H_2_O_2_ to induce cell injury in EA. hy926 cells for the study of endothelial injury including our previously published studies (9, 17, 38). The individual treatments of Cur and Bai showed dose-dependent improvement of cell survival in EA. hy926 cells, which were in line with the previous studies. Remarkably, the Cur+Bai combination has demonstrated a synergistic activity in restoring cell viability in EA. hy926 cells. Cur+Bai also reduced apoptotic level shown by the caspase-3 protein expression.

Further investigation on Cur+Bai combination in restoring the entire macrovascular pathological changes was conducted in a diabetic rat model. Two previous *in vivo* studies demonstrated that pre-treatment of Cur (15 days orally, 200 mg/kg or 2 weeks of 200 to 400 mg/kg, P.O.) prevented the increase in oxidative stress, reduced the cell apoptosis level and repaired the endothelial function as observed in the aortic tissue against cyclosporin A (31) and methotrexate (39). Bai has also been demonstrated to inhibit high glucose induced vascular inflammation in mice (13) and STZ-induced diabetic retinopathy rats (40). In this study, Cur and Bai used alone significantly lowered the FBG level, and reduced the blood lipids levels including TG, LDLC and NEFA, compared to the diabetic model group. Remarkably, the effect of the Cur+Bai combination was the greatest among all treatments that showed the lowest levels of FBG and tested blood lipids. It is worth mentioning that the dosage of Cur and Bai in the individual treatment group (150 mg/kg) doubled the dosage of Cur and Bai in the combination group (75 mg/kg for each), but the Cur+Bai generally showed a comparable or even greater effect in lowering the blood lipid levels. We then focused on the effect of Cur+Bai in protecting morphology and function of blood vessel in the diabetic model. The Cur+Bai combination significantly restored the eNOS protein expression, suggesting a remarkable protective effect on endothelial function in the diabetic rats. The pathological staining showed that the Cur+Bai combination maintained the normal structure of the endothelium and reduced adipose tissue in comparison to that of the diabetic model group. Thus, the endothelial protective action of Cur+Bai may partially be attributed to its anti-hyperglycemic and anti-hyperlipidemic activities. The TUNEL staining showed a significantly lower level of cell loss in the Cur+Bai treatment group compared to that of the model group in the three layers of the blood vessel, indicating its protection of blood vessel against diabetic angiopathy *in vivo*. Taken together, the above-mentioned results supported the feasible use of Cur+Bai as an oral supplement treatment (once a day) for diabetic angiopathy with a short treatment duration to regulate the glucose metabolism and restore the blood vessel status and function in diabetic angiopathy. The clinical dosage used in this study for Cur+Bai is estimated to be 1.5 g per 60 kg body weight, which is consisted of 0.75 g Cur and 0.75 g Bai. The clinical dosage used for Cur alone to improve the symptoms of diabetic microangiopathy is approximately 1 g per day for 4 weeks (41). In this case, if Cur is prepared in the Cur+Bai combination, a greater effect maybe reached with only half of the dosage. Further clinical trials are warranted to confirm the assumption.

Although there is an increasing number of studies that showed synergistic activity of phytochemicals (14), understanding the molecular mechanisms of synergy remains a challenge as it usually involves multi-faceted molecular targets. Previous studies have revealed the possible molecular mechanisms of Cur and Bai as individual compounds. Sun etal. (32) has linked the antioxidant action of Cur in protecting endothelium to the restored enzymatic activity of sirtuin 1 (SIRT1), which then upregulated the phosphorylation of eNOS and nitric oxide (32). The effect of Bai on diabetic-induced endothelial dysfunction was related to both antioxidant and anti-inflammatory activities (4, 13, 40). Bai was shown to inhibit the activation of nuclear factor (NF)-κB, the central signaling pathway of inflammation. In turn, it attenuated the vascular inflammatory response which is a critical event underlying the development of diabetic angiopathy (13).

In the present study, the molecular mechanisms of Cur and Bai, and their combination were investigated through a network pharmacology analysis. Our results revealed that (1) there are a number of overlapping genes and KEGG pathways (common targets) that are mediated by Cur and Bai against endothelial dysfunction, (2) top commonly targeted genes included MAPK 9, 10 and 14, and top targeted KEGG pathways included Pathways in cancer, Endocrine resistance and Fluid shear stress and atherosclerosis. The common gene targets and the KEGG pathway might be the key to explore the mechanisms of action of the Cur+Bai combination. The importance of the MAPK pathway was emphasized by the genes MAPK 8, MAPK10 and MAPK 14, which were all identified as common targets of Cur and Bai. The Nrf2 pathway was investigated due it is role as the essential antioxidant switcher to initiate the anti-atherosclerosis response in the Fluid sheer stress and atherosclerosis pathway. Additionally, MAPKp38 and JNK are essential protein kinases that participates in the pro-atherogenic response in the Fluid sheer stress and atherosclerosis pathway. Thereby these molecular targets are focused for the mechanistic study on Cur+Bai against diabetic angiopathy.

We demonstrated that the protective effect of the Cur+Bai combination in EA. hy926 cells was associated with Nrf2-HO-1 mediated antioxidant defense system. This was evidenced by significantly inhibited ROS level, magnified Nrf2 luciferase, upregulated HO-1 protein expressions as well as increased Phase II antioxidant enzymes including SOD and NAD enzymatic activities. Furthermore, the up-regulatory activities on Nrf2, HO-1 and phase II antioxidant enzymes by Cur+Bai were all significantly higher than that of Cur or Bai alone, which may help to explain the observed synergistic activity of Cur+Bai in restoring the impaired cell viability. Nrf2 is a master regulator of cellular resistance to oxidants which controls the basal and induced expression of an array of antioxidant response element-dependent genes (42). The induction of Nrf2 launches the expressions of Phase II antioxidant enzymes such as SOD and total NAD+ that directly detoxify ROS and thus reduce the level of oxidative stress (43–45). In fact, the results from our previous study demonstrated a synergistic activity of Cur and resveratrol in attenuating H_2_O_2_-induced endothelial oxidative damage and apoptosis, and the synergistic mechanism was associated with further strengthened upregulation of Nrf2-HO-1 mediated antioxidant pathway (9). Thus, it was speculated that this finding could be used to identify novel synergistic combination. Indeed, our present study showed that Cur interacted with Bai synergistically which might be partially related to the further upregulated Nrf2-HO-1 pathway. This finding underpins our previous finding that synergy may occur through the strengthening of the Nrf2-HO-1 pathway.

The prediction of network pharmacology on the synergistic mechanism of the Cur and Bai combination has been partially confirmed by our experimental investigation on the protein kinases of MAPKp38 and JNK. Our *in vitro* investigation showed that the phosphorylated p38 and JNK increased significantly by the stimulation of H_2_O_2_, which are key regulators in oxidative stress and inflammatory response in endothelial apoptosis (46, 47). The Cur+Bai combination significantly downregulated the protein kinase of p38 and JNK which the effect was greater than Cur or Bai alone. This finding supported the synergistic action of Cur+Bai in reducing cell apoptosis in EA. hy926 cells. In addition, the downregulation of p38 and JNK by Cur+Bai in diabetic rats may contribute to its observed protective effect on blood vessel. These experimental findings partially confirmed our network pharmacology results, although additional molecular targets and their crosstalk may also be involved, warranting further investigation and validation. Our results showed that the Cur+Bai combination also effectively reduced the level of TNF-α in the blood vessel tissue, the production of which is associated with the Fluid shear stress and atherosclerosis KEGG pathway and pathological progression of diabetic angiopathy (48).

Extrapolation of the results from cellular-based *in vitro* study to that of an *in vivo* investigation still present a great challenge. Some pharmacokinetics processes such as absorption, distribution, metabolism and elimination need to be taken into account when the efficacy of a phytochemical in a whole organism is concerned. Cur has been confirmed to exhibit very poor oral bioavailability (around 1%) (49) which rendered undetectable or extremely low amount in blood (50). Orally administered curcumin is transformed into dihydrocurcumin and tetrahydrocurcumin within one hour, and then largely converted into glucuronide and glucuronide/sulfate conjugates (51). Thus, the observed effect of Cur in the diabetic rats may be attributed to the functional role of these metabolites. Tetrahydrocurcumin, the main metabolite of Cur in the blood circulation, was shown to suppress oxidative stress (52), and improved vascular dysfunction, arterial stiffness, and hypertension associated with cadmium exposure in mice (53). The effect of glucuronide and glucuronide/sulfate conjugates in protecting vascular function against oxidative stress is unknown. Oral Bai also undergoes a fast and extensive phase II metabolism with a negligible absorption in rats (54, 55). Bai via oral administration was mainly transformed into its conjugated metabolites, glucuronides/sulfates of baicalein (75.7%) (56) including baicalin which were extensively circulating in the plasma (57, 58) (54). Baicalin is shown to repair the blood vessel function in aorta in diabetic mice, due to a mechanism associated with Nrf2 signaling (4). Thus, the observed effect of Bai in this study may at least partly be attributable to the action of baicalin. Thereby, the underlying mechanism of the combined action of Cur+Bai in restoring on macrovascular changes may rely on the action of their metabolites. The next step can be the pharmacokinetic study of Cur+Bai in the diabetic rats and determination of the ratio of their metabolites, followed by the study of the interaction of their metabolites.

At present, there is no robust statistical analysis method available to quantify synergy in a combination at one dosage level. Thus the “synergy” in this study was based on statistical comparison between the individual treatments to that of the combined treatments (i.e. t-test or one-way analysis of variance). There are several other limitations of the study. The secondary cell line, EA. hy926 cells were used to investigate the protective effect of the Cur+Bai combination in endothelial cells, rather than using the primary aortic endothelial cells, which does not fully inform the effects of Cur+Bai on native endothelium in aortic blood vessels. The effect of Cur+Bai observed in the animal study was only based on male animals, but not on female’s counterparts. The quantified phenotype changes in the endothelium following *in vivo* treatment were limited in this study. Due to the absence of pharmacokinetic data, there remains challenge to investigate the Cur+Bai combination as a therapeutic agent in the management of diabetic angiopathy.

## Conclusions

The presented study suggested that Cur and Bai interacted synergistically in restoring cell survival in H_2_O_2_ impaired EA. hy926 cells. The combined treatment of Cur and Bai reduced FBG and blood lipids levels including TCHO, TG, LDLC and NEFA in diabetic GK rats. The Cur+Bai combination protected the endothelium function, maintained the blood vessel wall and reduced the adipose tissue in both aortic arch and mid thoracic aorta blood vessels.

The mechanisms associated with the synergistic interactions of Cur and Bai were investigated by the network pharmacology analysis. MAPK gene targets and Fluid sheer stress and atherosclerosis pathway were analysed to be the key common targets of Bai and Cur against endothelial dysfunction. Our *in vitro* results revealed that the Cur+Bai combination reduced ROS fold change and upregulated Nrf2, HO-1, SOD, and NAD regulators. It also markedly downregulated the phosphorylation of MAPKp38 and JNK against the stimulation of H_2_O_2_. In diabetic rats, the Cur+Bai combination upregulated the protein expressions of Nrf2 and HO-1, as well as increased the enzymatic activities of SOD, NAD+/NADPH, and decreased the enzymatic level of MPO. Furthermore, it inhibited the protein kinases of p38 and JNK. Cur and Bai also significantly reduced the level of TNF-α in the blood vessel tissue.

Altogether, our results demonstrated that the Cur+Bai combination significantly attenuated endothelial injury against oxidative damage in endothelial cells and effectively protected blood vessel in diabetic rats via regulating Nrf2-HO-1 mediated antioxidant defensive system and MAPK pathways. Our findings may help develop novel combination therapies with multi-targeted actions for mitigating endothelial dysfunction in diabetic angiopathy against hyperglycaemia, hyperlipidaemia and oxidative damage.

## Data availability statement

The original contributions presented in the study are included in the article/**Supplementary Material**. Further inquiries can be directed to the corresponding authors.

## Ethics statement

The animal study was reviewed and approved by the Animal Care and Use Committee of Fujian University of Traditional Chinese Medicine.

## Author contributions

Conceptualization, XZ and YFZ; methodology, CW, YS and WL; software, SL, YLu, JG and XZ; validation, JC, CL and DC; formal analysis, YPZ, ZC and YXL; investigation, CW, YS, WL, SA and XZ; resources, CL, YFZ, and MH; data curation, JC., ZC, YXL and YPZ; writing original draft preparation, CW and XZ; writing review and editing, XZ, YFZ, DC, CL and GM; visualization, XZ and YFZ; supervision, YFZ, XZ and MH; project administration, XZ, YFZ and MH; funding acquisition, XZ, YFZ and MH. All authors contributed to the article and approved the submitted version.

## Funding

This project is supported by the ICON (Improving Cardiovascular Outcome Network) Early Career Researchers Program awarded to XZ from the South Western Sydney Local Health District. The project is also funded by the National Natural Science Foundation of China (81973437, 81703909), the Fujian Provincial Marine Economic Development Special Fund Project (FJHJF-L-2021-4), the Collaborative Innovation Platform Project of Fuxiaquan National Innovation Demonstration Zone (2021FX02). XZ is supported by the Research Support Program Fellowship, Western Sydney University.

## Acknowledgments

We thank to Mr John Truong for his kind support.

## Conflict of interest

The authors declare that the research was conducted in the absence of any commercial or financial relationships that could be construed as a potential conflict of interest.

As a medical research institute, NICM Health Research Institute receives re-search grants and donations from foundations, universities, government agencies, individuals and industry. Sponsors and donors also provide untied funding for work to advance the vision and mission of the Institute.

## Publisher’s note

All claims expressed in this article are solely those of the authors and do not necessarily represent those of their affiliated organizations, or those of the publisher, the editors and the reviewers. Any product that may be evaluated in this article, or claim that may be made by its manufacturer, is not guaranteed or endorsed by the publisher.
