# Supplementary material for: Protective effect of the curcumin-baicalein combination against macrovascular changes in diabetic angiopathy
Source: Front Endocrinol (Lausanne). 2022 Aug 18;13:953305. doi: 10.3389/fendo.2022.953305 (PMC9433877; doi:10.3389/fendo.2022.953305)
Supplement: Supplementary file 1 [file DataSheet_1.pdf]

Supplementary Material

1 Supplementary Data

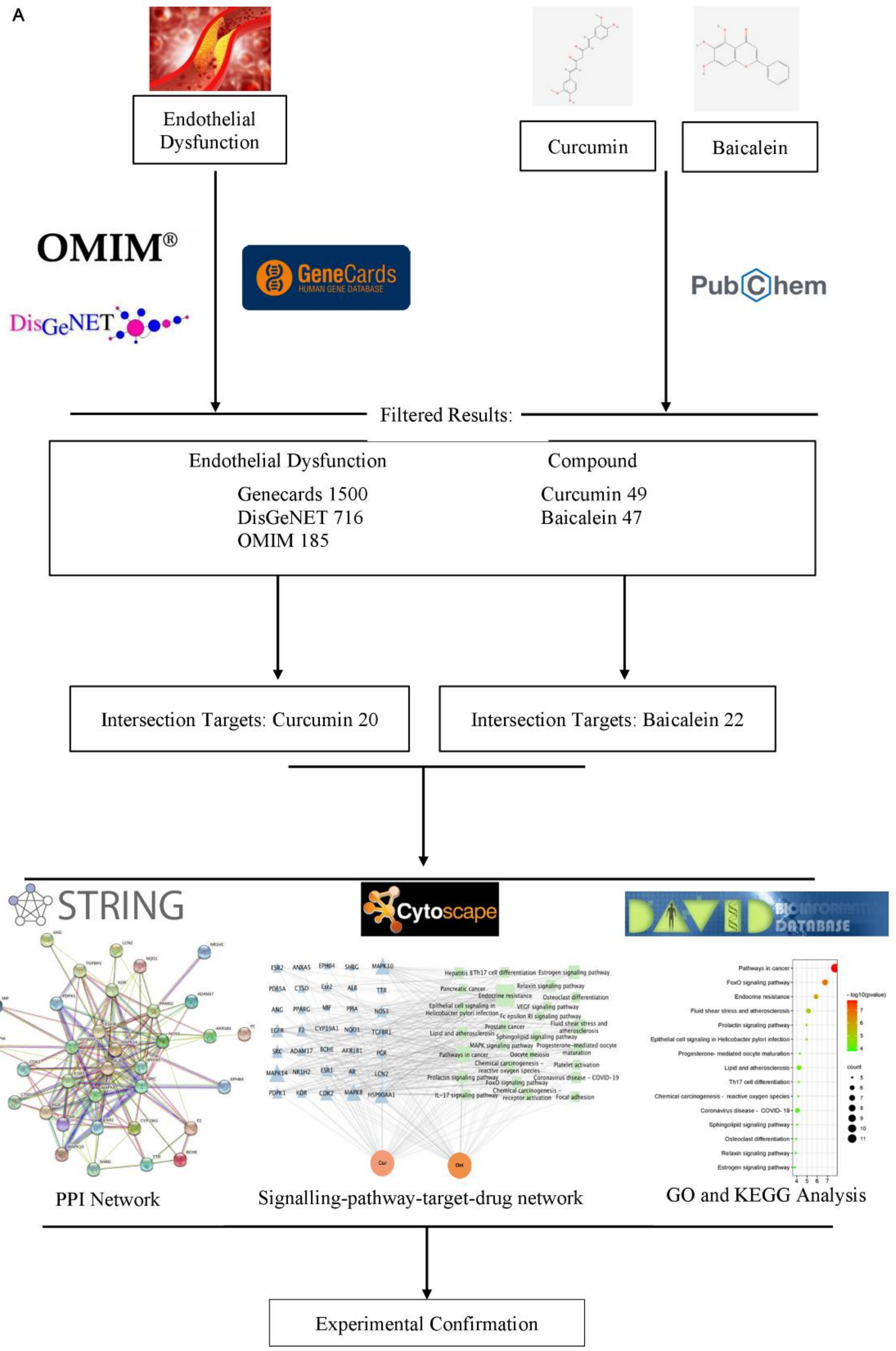

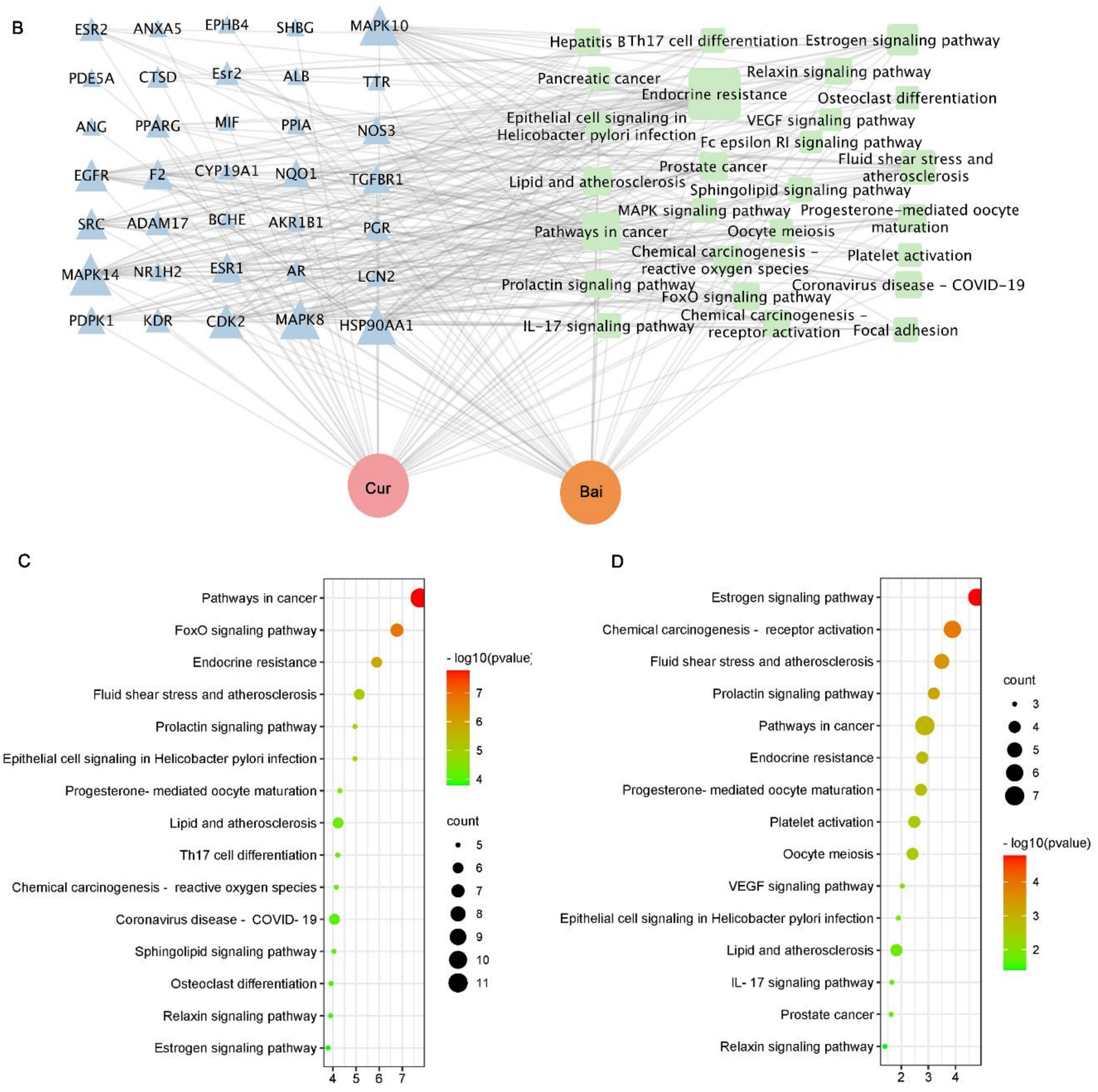
B
ESR2
ANXA5
EPHB4
SHBG
MAPK10
PDE5A
CTSD
Esr2
ALB
TTR
ANG
PPARG
MIF
PPIA
NOS3
EGFR
F2
CYP19A1
NQO1
TGFBR1
SRC
ADAM17
BCHE
AKR1B1
PGR
MAPK14
NR1H2
ESR1
AR
LCN2
PDPK1
KDR
CDK2
MAPK8
HSP90AA1
Hepatitis B
Th17 cell differentiation
Estrogen signaling pathway
Relaxin signaling pathway
Pancreatic cancer
Endocrine resistance
Osteoclast differentiation
Epithelial cell signaling in Helicobacter pylori infection
VEGF signaling pathway
Fc epsilon RI signaling pathway
Prostate cancer
Fluid shear stress and atherosclerosis
Lipid and atherosclerosis
Sphingolipid signaling pathway
MAPK signaling pathway
Progesterone−mediated oocyte maturation
Pathways in cancer
Oocyte meiosis
Chemical carcinogenesis − reactive oxygen species
Platelet activation
Prolactin signaling pathway
Coronavirus disease − COVID−19
FoxO signaling pathway
IL−17 signaling pathway
Chemical carcinogenesis − receptor activation
Focal adhesion
Cur
Bai
C
Pathways in cancer
FoxO signaling pathway
Endocrine resistance
Fluid shear stress and atherosclerosis
Prolactin signaling pathway
Epithelial cell signaling in Helicobacter pylori infection
Progesterone- mediated oocyte maturation
Lipid and atherosclerosis
Th17 cell differentiation
Chemical carcinogenesis - reactive oxygen species
Coronavirus disease - COVID- 19
Sphingolipid signaling pathway
Osteoclast differentiation
Relaxin signaling pathway
Estrogen signaling pathway
4 5 6 7
- log10(pvalue)
7
6
5
4
count
5
6
7
8
9
10
11
D
Estrogen signaling pathway
Chemical carcinogenesis - receptor activation
Fluid shear stress and atherosclerosis
Prolactin signaling pathway
Pathways in cancer
Endocrine resistance
Progesterone- mediated oocyte maturation
Platelet activation
Oocyte meiosis
VEGF signaling pathway
Epithelial cell signaling in Helicobacter pylori infection
Lipid and atherosclerosis
IL- 17 signaling pathway
Prostate cancer
Relaxin signaling pathway
2 3 4
count
3
4
5
6
7
- log10(pvalue)
4
3
2

E

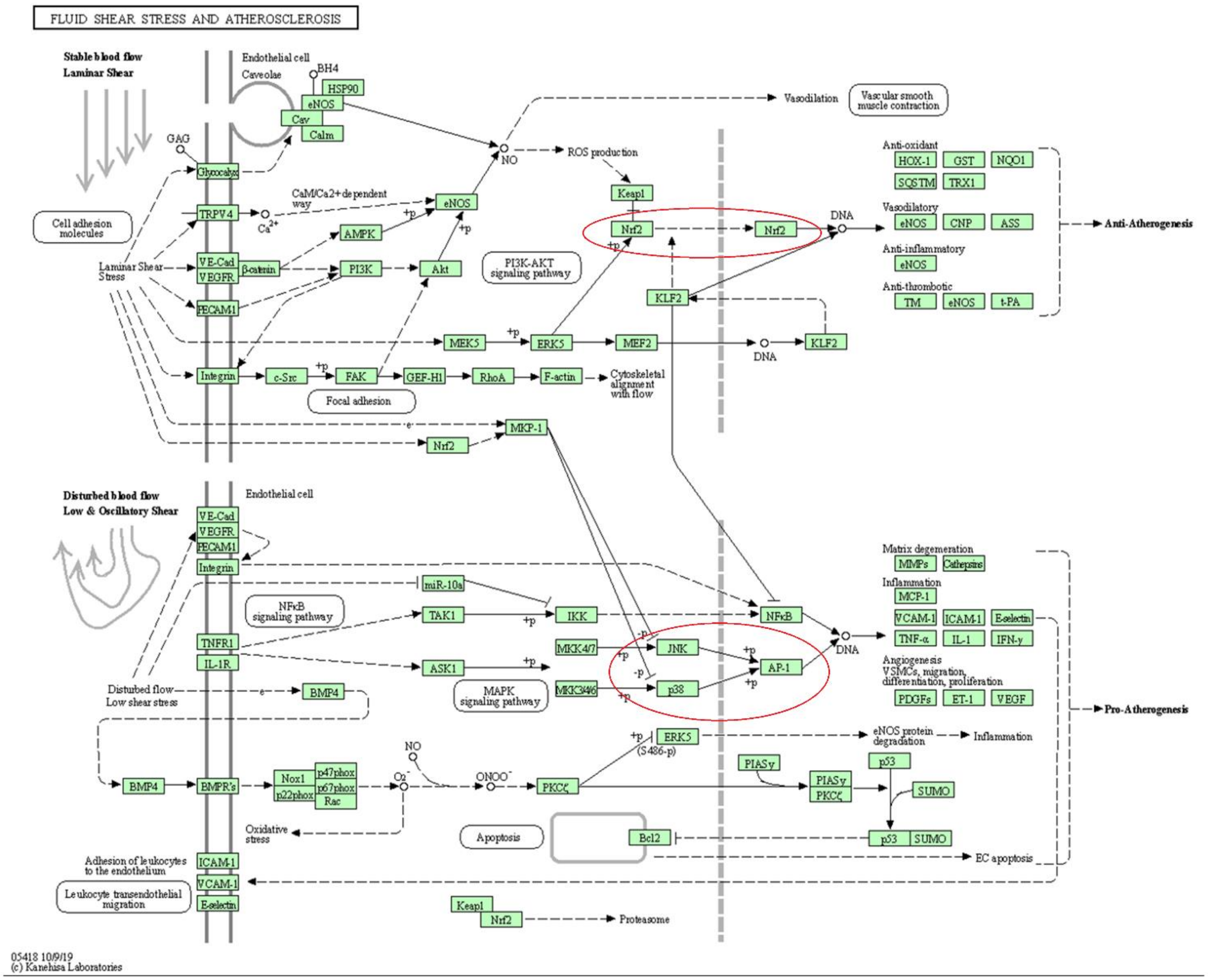

Supplementary Figure 1. Multi-target-multi-pathway network of Cur and Bai on treating endothelial dysfunction. (A) Workflow of the construction of the PPI network, signaling pathway-target-drug network, and GO/KEGGE analysis of Cur and Bai. (B) There were 35 differentially expressed genes which were validated in published literatures. This network depicted a clear multi-targeted pathways through modulating groups of genes to show their pharmacological and clinical effects. Compound was linked to gene targets, while gene targets were linked to pathways. Circle represents compounds; Triangle represents target genes; Square represents related KEGG pathways. (C) Top 15 KEGG pathways relevant to the action of Cur against endothelial dysfunction. (D) Top 15 KEGG pathway relevant to the action of Bai against endothelial dysfunction. (E) Fluid shear stress and atherosclerosis KEGG pathway (ID: 05418109/19) obtained from Kanehisa Laboratories (Kanehisa, 2019; Kanehisa et al., 2021; Kanehisa et al., 2000). Red circles highlight the key regulator in the KEGG pathway shown in the map.

A

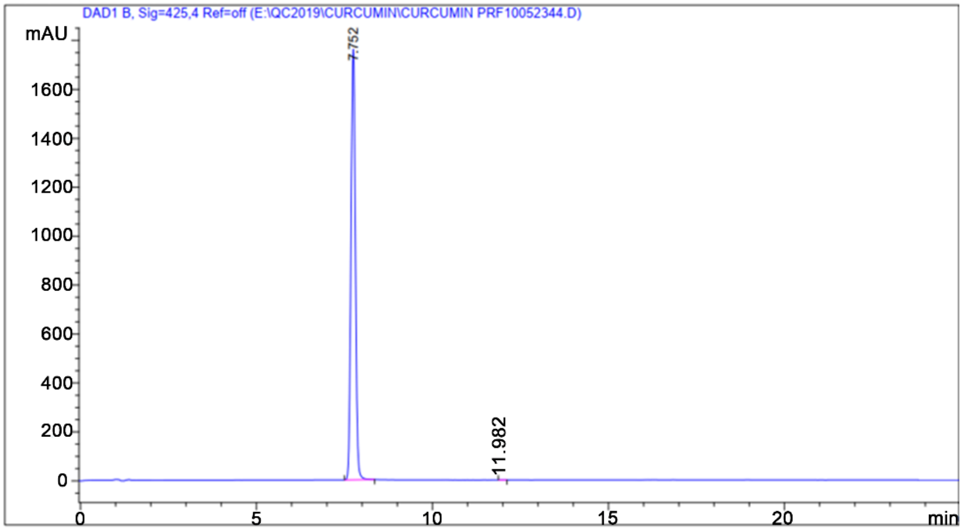

Peaks	Retention time (min)	Peak area (mAU*s)	Peak height	Peak area (%)
1	7.752	1.53921e4	1762.85742	**99.9842**
2	11.982	2.42821	3.23029e-1	0.0158

B

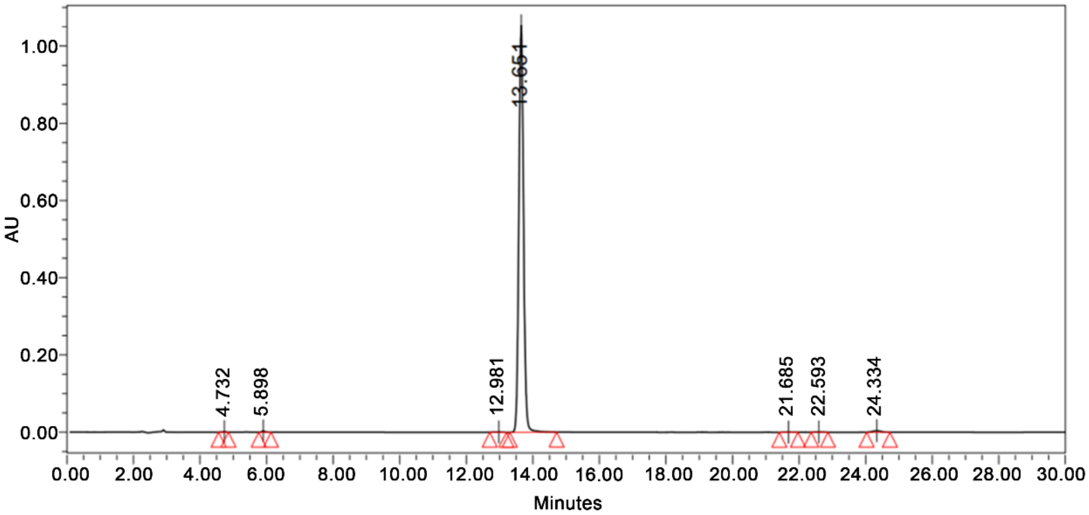

Peaks	Retention time (min)	Peak area (mAU*s)	Peak height (µV)	Peak area (%)
1	4.732	9788	1624	0.09
2	5.898	22093	3453	0.21
3	12.981	13427	984	0.13
4	13.651	10351271	1052992	**98.63**
5	21.685	8895	620	0.08
6	22.593	15742	1076	0.15
7	24.334	73520	4204	0.70

Supplementary Figure 2. The identity and purity of the chemicals, curcumin and baicalein, used in this study. HPLC fingerprint and peak heights/areas of (A) Cur (peak 1) and (B) Bai (peak 4) used in the study. The purity of them were both higher than 98% as quantified by their peak area percentages.

A

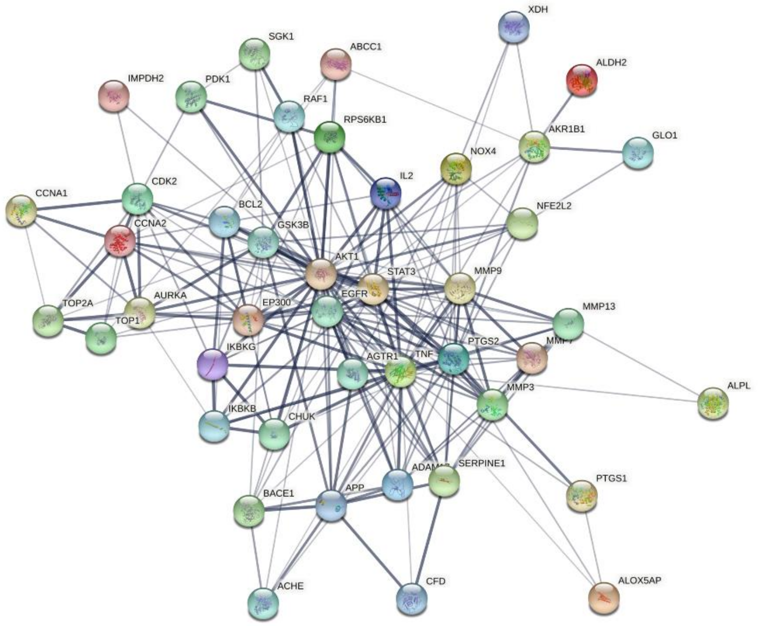

B

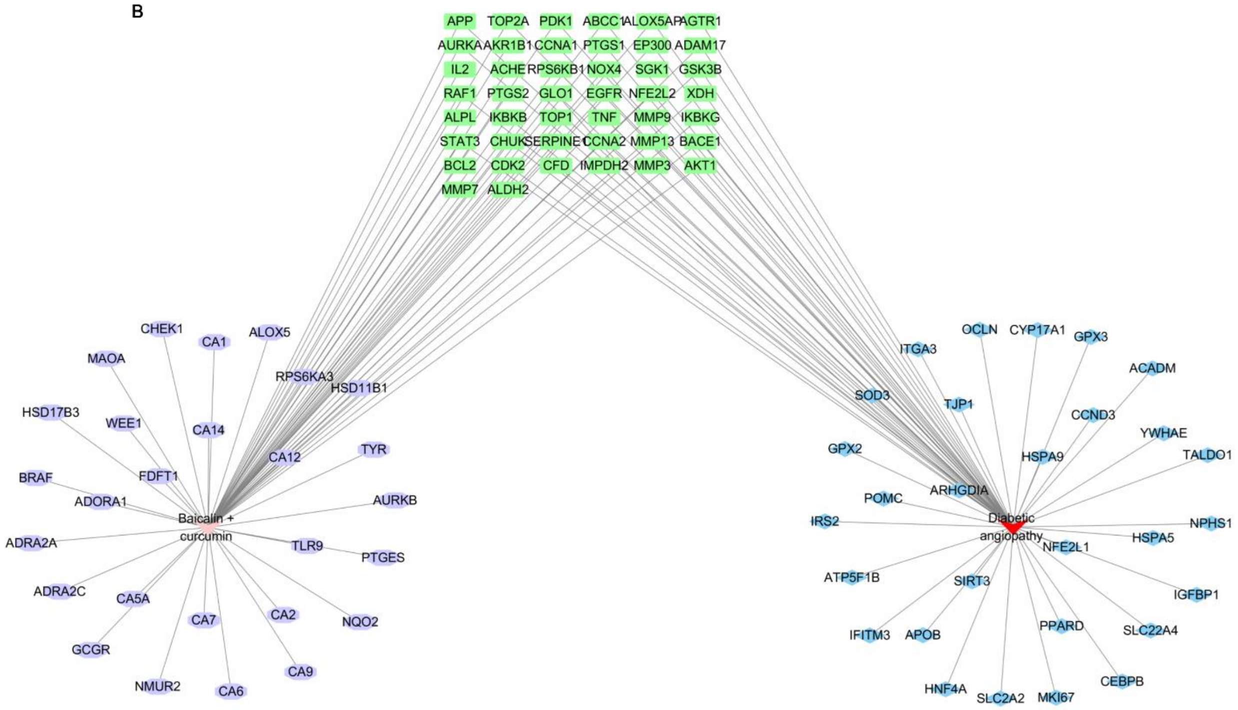

C

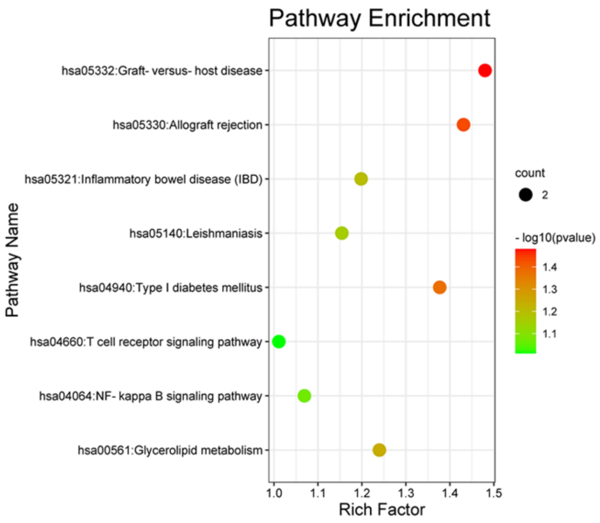

Supplementary 2D

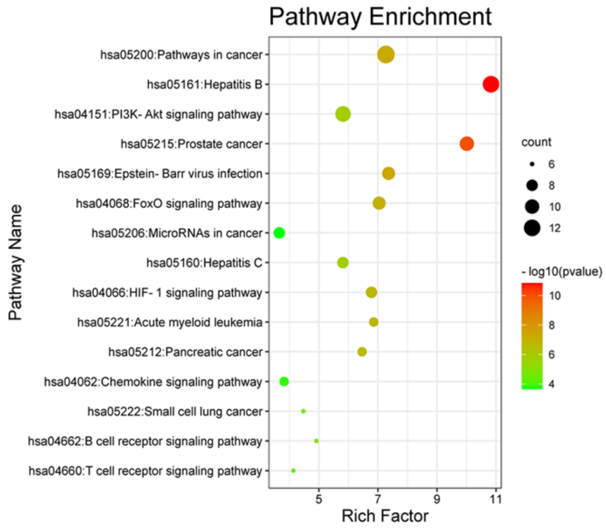

Supplementary Figure 3. Gene, protein targets and associated KEGG pathways of curcumin (Cur) and baicalein (Bai) against diabetic angiopathy as analysed by network pharmacology. (A) PPI network, (B) compounds-genes-KEGG pathways network constructions, top 15 associated KEGG pathways of Cur (C) and Bai (D) against diabetic angiopathy.

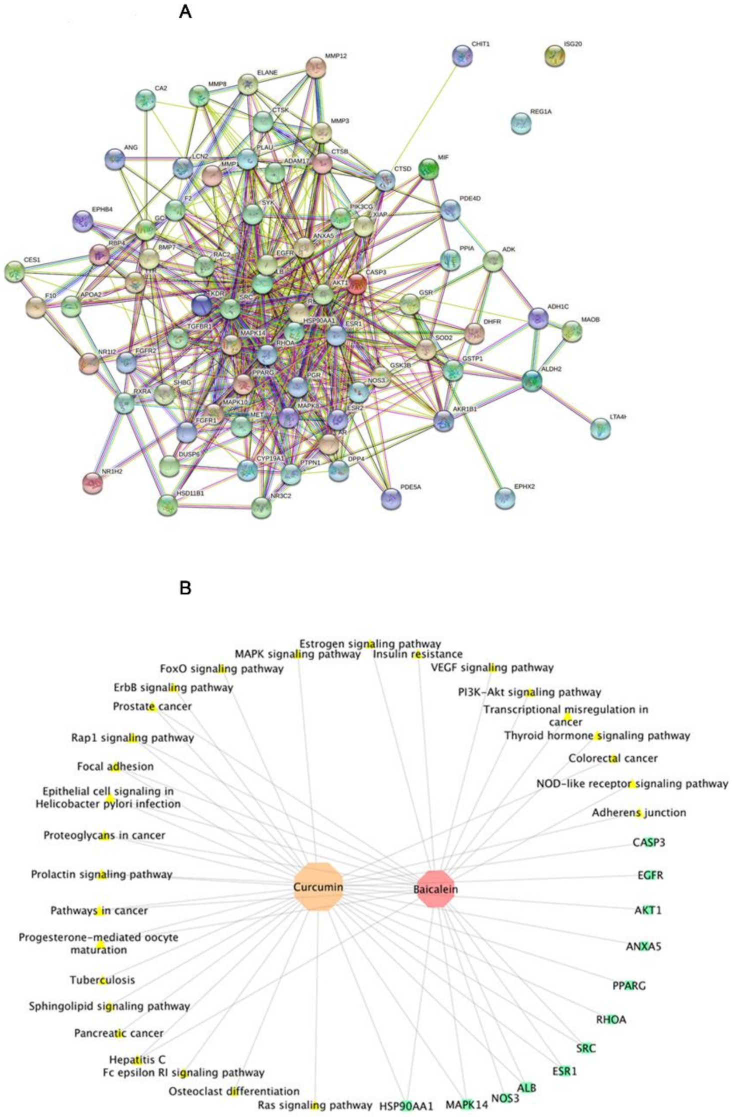
A
B
Estrogen signaling pathway
MAPK signaling pathway
Insulin resistance
FoxO signaling pathway
VEGF signaling pathway
ErbB signaling pathway
PI3K-Akt signaling pathway
Prostate cancer
Transcriptional misregulation in cancer
Rap1 signaling pathway
Thyroid hormone signaling pathway
Colorectal cancer
Focal adhesion
NOD-like receptor signaling pathway
Epithelial cell signaling in Helicobacter pylori infection
Adherens junction
Proteoglycans in cancer
CASP3
Prolactin signaling pathway
Curcumin
Baicalein
EGFR
Pathways in cancer
AKT1
Progesterone-mediated oocyte maturation
ANXA5
Tuberculosis
PPARG
Sphingolipid signaling pathway
RHOA
Pancreatic cancer
SRC
Hepatitis C
ESR1
Fc epsilon RI signaling pathway
ALB
Osteoclast differentiation
NOS3
Ras signaling pathway
HSP90AA1
MAPK14

C

Supplementary 3D

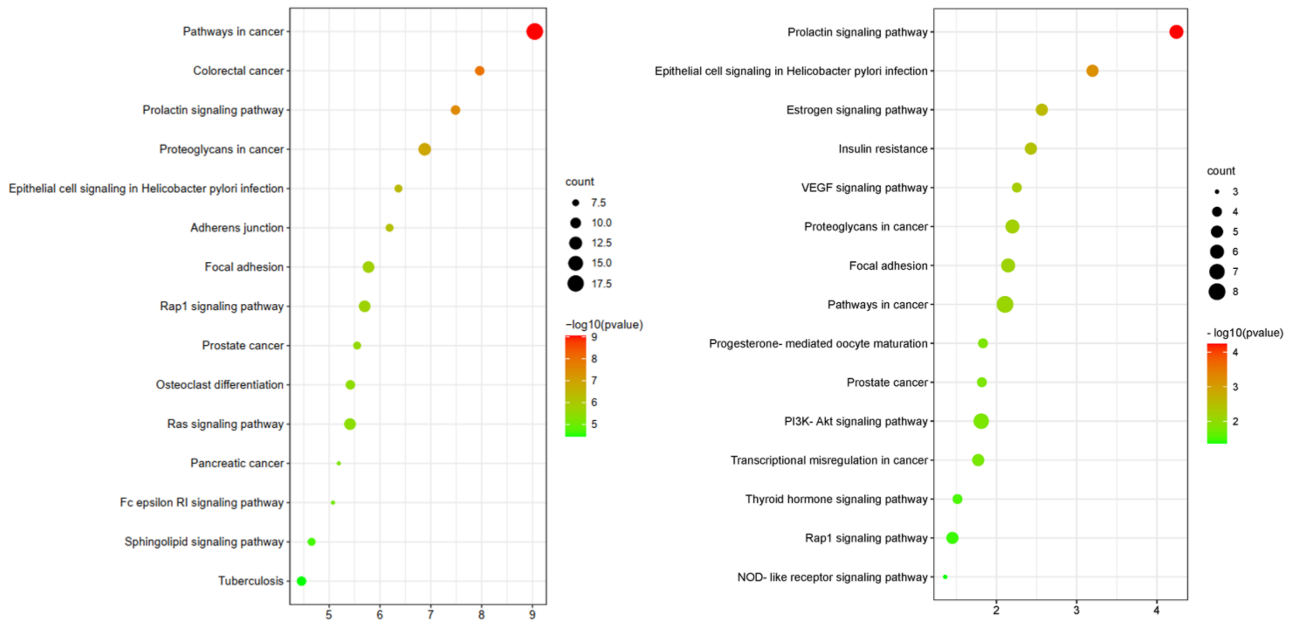

Supplementary Figure 4. Gene, protein targets and associated KEGG pathways of curcumin (Cur) and baicalein (Bai) against atherosclerosis and endothelial dysfunction as analysed by network pharmacology. (A) PPI network, (B) compounds-genes-KEGG pathways network constructions, top 15 associated KEGG pathways of Cur (C) and Bai (D) against atherosclerosis/endothelial dysfunction.
